# Bias estimation in study design: a meta-epidemiological analysis of transcatheter versus surgical aortic valve replacement

**DOI:** 10.1186/s12893-021-01278-0

**Published:** 2021-06-07

**Authors:** Saerom Youn, Shannon Avery Wong, Caitlin Chrystoja, George Tomlinson, Harindra C. Wijeysundera, Chaim M. Bell, Anna R. Gagliardi, Nancy N. Baxter, Julie Takata, Lakhbir Sandhu, David Robert Urbach

**Affiliations:** 1 Institute of Health Policy, Management, and Evaluation, University of Toronto, Toronto, ON Canada; 2 Schulich School of Medicine and Dentistry, University of Western Ontario, London, ON Canada; 3 College of Medicine and Dentistry, James Cook University, Parkville, QLD Australia; 4 Department of Medicine, University of Toronto, Toronto, Canada; 5 Department of Surgery, University of Toronto, Toronto, Canada; 6 Li Ka Shing Knowledge Institute, St. Michael’s Hospital, Toronto, ON Canada; 7 School of Population and Global Health, University of Melbourne, Melbourne, VIC Australia; 8 Women’s College Hospital Research Institute (WCRI), Toronto, ON Canada; 9 Department of Surgery, Women’s College Hospital, 76 Grenville St, Room 8332, M5S 1B2, Toronto, ON Canada; 10 Women’s College Hospital Institute for Health System Solutions and Virtual Care (WIHV), Toronto, ON Canada

**Keywords:** Non drug health technologies, Randomized controlled trials, Nonrandomized studies, Bias, Study design attributes, Meta-epidemiological, Meta-regression, Aortic stenosis, TAVI, SAVR

## Abstract

**Background:**

Paucity of RCTs of non-drug technologies lead to widespread dependence on non-randomized studies. Relationship between nonrandomized study design attributes and biased estimates of treatment effects are poorly understood. Our purpose was to estimate the bias associated with specific nonrandomized study attributes among studies comparing transcatheter aortic valve implantation with surgical aortic valve replacement for the treatment of severe aortic stenosis.

**Results:**

We included 6 RCTs and 87 nonrandomized studies. Surgical risk scores were similar for comparison groups in RCTs, but were higher for patients having transcatheter aortic valve implantation in nonrandomized studies. Nonrandomized studies underestimated the benefit of transcatheter aortic valve implantation compared with RCTs. For example, nonrandomized studies without adjustment estimated a higher risk of postoperative mortality for transcatheter aortic valve implantation compared with surgical aortic valve replacement (OR 1.43 [95% CI 1.26 to 1.62]) than high quality RCTs (OR 0.78 [95% CI 0.54 to 1.11). Nonrandomized studies using propensity score matching (OR 1.13 [95% CI 0.85 to 1.52]) and regression modelling (OR 0.68 [95% CI 0.57 to 0.81]) to adjust results estimated treatment effects closer to high quality RCTs. Nonrandomized studies describing losses to follow-up estimated treatment effects that were significantly closer to high quality RCT than nonrandomized studies that did not.

**Conclusion:**

Studies with different attributes produce different estimates of treatment effects. Study design attributes related to the completeness of follow-up may explain biased treatment estimates in nonrandomized studies, as in the case of aortic valve replacement where high-risk patients were preferentially selected for the newer (transcatheter) procedure.

**Supplementary Information:**

The online version contains supplementary material available at 10.1186/s12893-021-01278-0.

## Background

Frameworks of study designs often specify hierarchies based on the likelihood of estimating biased treatment effects, with well-designed randomized controlled trials (RCT) and their meta-analyses considered to provide the least biased estimates [1–3]. However, there are limited RCTs of non-drug technologies such as medical devices and surgical techniques [4, 5], leading to widespread dependence on non-randomized studies for the evaluation of non-drug health technologies.

Not surprisingly, there is variation in the treatment effects estimated by different study designs, with non-randomized studies frequently reporting larger benefits for the experimental treatment than RCTs [6–12]. Differences in the conclusions of non-randomized studies and RCTs vary according to the clinical context [13–16]. Among RCTs, study quality is associated with estimates of treatment effects; lower quality RCTs often overestimate the benefit of an experimental procedure as compared to high quality RCTs [17–21]. The relationship between study attributes and biased treatment effects is less clear for nonrandomized studies—a better understanding of this relationship would help inform readers, providers, patients, and policy makers, especially when data from high-quality RCTs are not available.

There are many nonrandomized studies and RCTs comparing transcatheter and surgical aortic valve replacement for the treatment of aortic stenosis, providing an ideal opportunity to study the influence of study designs and characteristics on estimates of treatment effectiveness. We sought to empirically explore the direction and magnitude of bias associated with different study attributes using a meta-epidemiological analysis of published studies.

## Methods

The study was performed in accordance with the PRISMA guidelines for meta-epidemiological studies [22]. A summary flow chart of research methodology is available in Additional file 1: Figure S1.

### Clinical context

We studied transcatheter and surgical aortic valve replacement for aortic stenosis because there were both high quality RCTs and a large number of non-randomized studies. Transcatheter aortic valve implantation is a relatively new technique, and its safety and efficacy is of current clinical interest.

### Study selection

We included all RCTs that randomly assigned patients to transcatheter or surgical aortic valve replacement and followed patients over time. We also included all comparative cohort studies that reported primary data on outcomes of interest after transcatheter or surgical aortic valve replacement.

We excluded non-randomized studies that were not comparative cohort studies, defined the population by excluding the outcome of interest, combined patients from RCTs and non-randomized studies, conference abstracts, poster presentations, non-peer reviewed publications, unpublished literature, systematic reviews that lacked primary data, and studies that used other surgical aortic valve replacement methods (e.g., minimally invasive, sutureless).

For multiple publications using the identical cohort we included the publication with the most representative sample, determined by sample size or duration of follow up.

### Data sources

We searched Medline, Medline In-Process/ePubs, Embase, Cochrane Central Register of Controlled Trials, Cochrane Database of Systematic Reviews, Scopus, and Web of Science from inception to June 2017 (Additional file 1: Table S1). We used DistillerSR (Evidence Partners, Ottawa, Canada) to check for duplicate citations, and to screen titles, abstracts, and full text.

### Data extraction

A single reviewer collected study characteristics, patient characteristics, and outcomes of interest; questions were resolved by consensus among the study team. Agreement of re-abstracted outcomes for a sample of 15 nonrandomized studies (17%) by a second reviewer demonstrated excellent inter-rater reliability (ICC 0.99 [95% CI 0.98 to 0.99]) [23].

### Study characteristics

We collected study sample size, publication year and country, surgical approach, and the study time period. We collected surgical risk scores (e.g., EuroSCORE II) as a measure of potential selection bias among comparison groups.

### Outcomes

We defined postoperative mortality as death due to any cause within 1-month or in hospital after the procedure regardless of location. We defined length of stay as the number of days the patient stayed in the hospital after the procedure. We extracted the necessary components of each outcome to calculate the pooled estimates of treatment effects. We calculated missing data points using given information where possible.

### Explanatory variables: study designs

We categorized studies into 8 groups according to study design: (1) All (all RCT and nonrandomized studies), (2) All RCT, (3) High quality RCT, (4) Low quality RCT, (5) All non-randomized studies, (6) Nonrandomized studies without adjustment, (7) Nonrandomized studies adjusted using propensity score matching (PSM), and (8) Nonrandomized studies adjusted using regression.

RCTs were divided into high or low quality RCTs based on the Cochrane Risk Of Bias (ROB) tool [24] based on the content of the published articles; authors were not contacted for additional information. No RCT blinded study participants; hence RCTs that satisfied all other criteria were categorized as high quality. Non-randomized studies reported unadjusted estimates, adjusted estimates, or both. Non-randomized studies estimates were pooled into 3 groups: without adjustment, adjusted using PSM, and adjusted using regression.

Finally, we previously developed a set of 41 non-randomized studies attributes that could bias studies (Additional file 1: Table S2). These attributes were based on existing frameworks of bias and quality assessment tools for nonrandomized studies, and were extensively pilot tested and iteratively developed for clarity and reliability.

### Data synthesis

#### Study characteristics

We compared overall study and patient characteristics between RCTs and non-randomized studies using descriptive statistics. To combine continuous variables across studies, the weighted mean of estimates was calculated, and the pooled standard deviation (SD) was either calculated directly (where reported) or imputed from the pooled variance of included studies in the relevant group if missing [25].

### Pooled estimates of treatment effects

The effect of treatment on postoperative mortality was estimated using odds ratio (OR). OR < 1 indicated lower risk of death for transcatheter aortic valve implantation. For Bayesian RCTs, we assumed the median estimate represented the percentage with events [26, 27]. The treatment effect on length of stay was estimated using mean difference (MD, with values < 0 indicating shorter length of stay for transcatheter aortic valve implantation).

All effect sizes were pooled using a random effects model to account for potential between-study heterogeneity. For postoperative mortality, we used the DerSimonian-Laird method, [28] with the exception of estimates that incorporated adjusted ORs from nonrandomized studies adjusted using regression, which were calculated using the generic inverse variance method [25]. For length of stay, we used the inverse variance method [25]. All pooled estimates were presented visually using forest plots with point estimates and 95% CI. Estimates from high-quality RCTs were considered to represent the “gold standard” treatment effects.

We evaluated the impact of the 41 nonrandomized study attributes on estimates of treatment effect by calculating the ratio of odds ratios (ROR) for postoperative mortality and difference of mean differences (DMD) for length of stay with 95% CI using random effects meta regression. The ROR is the ratio of the OR in one group of studies and the OR in another group of studies [18]; the DMD is the difference between MD reported in one group of studies and the MD in another group of studies [29]. We compared the pooled estimates of the effect measures between study categories, and also between nonrandomized studies with attributes hypothesized to be associated with bias. In all comparisons, ROR < 1 and DMD < 0 indicated that studies favored transcatheter aortic valve implantation.

All statistical analyses were conducted using R studio version 1.0.136 (2016) [30]. The analysis of whether the attributes of nonrandomized studies were associated with statistical differences in pooled effect sizes was an exploratory analysis; a less restrictive 2-sided P value of 0.10 was used to determine potentially important attributes. In all other analyses a P value of 0.05 or less was considered statistically significant. P values for comparisons of estimates between types of study were those of the ROR or DMD for the comparison.

## Results

### Study selection

**Fig. 1:**
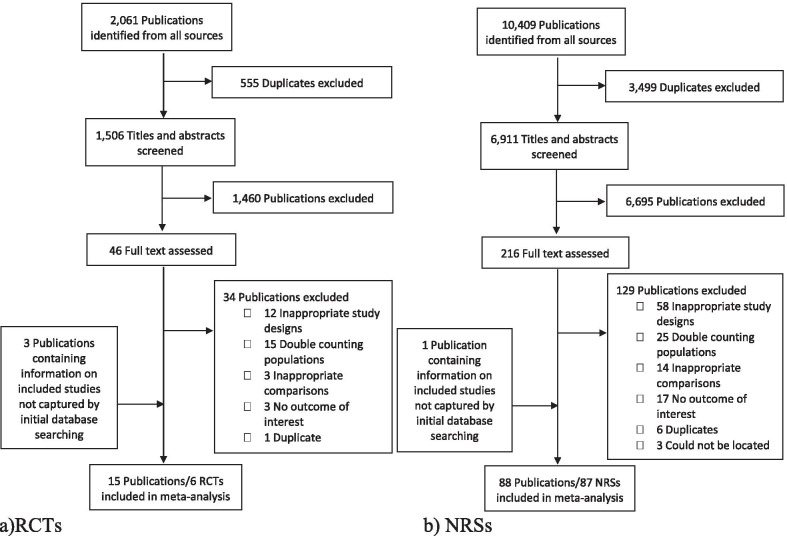
Flow diagram of literature search and screening to identify eligible studies. RCT, randomized controlled trials; NRS, nonrandomized studies

Of 2061 RCTs identified in our search, six (described in 15 publications) met the inclusion criteria, and of 10,409 nonrandomized studies, 87 (described in 88 publications) met the inclusion criteria (Fig. 1 and Additional file 1: Table S3). We included four additional nonrandomized studies from publications that were not identified in the initial search.

### Study characteristics

The six RCTs included 5352 patients, and the 87 non-randomized studies included 239,433 patients (Table 1). RCTs and nonrandomized studies had similar years of publication, were conducted mostly in Europe and North America, and often used multiple surgical approaches.

**Table 1:** Descriptive characteristics of included studies

	RCTs		NRSs	
Number of studies	6		87	
Total number of patients	5352		239,433	
Year published^a^	2014 (2012, 2016)		2014 (2012, 2016)	
Region				
Europe	2 (33.3%)		47 (54.0%)	
North America	2 (33.3%)		16 (18.4%)	
Asia	0		7 (8.0%)	
Other	0		3 (3.5%)	
Multiple	2 (33.3%)		2 (2.3%)	
Unclear	0		12 (13.8%)	
TAVI Approach				
Any	5 (83.3%)		57 (65.5%)	
Transfemoral	0		10 (11.5%)	
Transapical	1 (16.7%)		13 (14.9%)	
Other	0		7 (8.0%)	

	TAVI	SAVR	TAVI	SAVR
Number of patients	2771	2581	78,254	161,179
Year enrolment began	2010 (2008, 2011) (n = 6)	2010 (2008, 2011) (n = 6)	2009 (2006–2011) (n = 79)	2007 (2005–2009) (n = 76)
Year enrolment ended	2012 (2011, 2013) (n = 6)	2012 (2011, 2013) (n = 6)	2012 (2010–2013) (n = 75)	2012 (2010, 2013) (n = 72)
Baseline surgical risk^b^				
STS	6.13 ± 2.25 (n = 5)	6.20 ± 2.32 (n = 5)	9.83 ± 5.03 (n = 34)	6.76 ± 3.68 (n = 33)
EuroSCORE I	NA	NA	18.25 ± 8.61 (n = 8)	11.16 ± 5.26 (n = 8)
LogEuroSCORE	16.21 ± 8.77 (n = 5)	16.30 ± 8.64 (n = 5)	22.32 ± 11.29 (n = 44)	14.19 ± 8.73 (n = 44)
EuroSCORE II	NA	NA	8.52 ± 6.58 (n = 5)	8.09 ± 5.74 (n = 5)
NYHA	2.75 (n = 3)	2.74 (n = 3)	3.40 (n = 12)	2.62 (n = 12)

All continuous variables are reported as either median (25th, 75th percentile) or mean ± SD. All discrete variables are reported as n (%)

Values describing the characteristics of patients in each arm of the studies are followed by the number of studies each category that reported the value (n)

RCT, Randomized Controlled Trial; NRS, Nonrandomized Study; TAVI, Transcatheter Aortic Valve Implantation; SAVR, Surgical Aortic Valve Replacement; STS, Society of Thoracic Surgeons; NYHA, New York Heart Association; NA, Not Applicable

^a^For studies with multiple publications, the year of the first publication was used

^b^STS, EuroSCORE I, LogEuroSCORE and EuroSCORE II are measures of predicted operative mortality. NYHA classifies the extent of heart failure into 4 classes I to IV, with I being least severe and IV being most severe. The numbers indicate the weighted average NYHA class of each cohort. ‘Other’ TAVI approaches included non-iliofemoral, transthoracic, or transvascular approaches

The proportion of studies including patients of all surgical risk categories was higher in non-randomized studies than RCTs (67.8% vs 33.3%). In general, transcatheter aortic valve implantation subjects in nonrandomized studies had higher surgical risk compared to transcatheter aortic valve implantation subjects in RCTs or surgical aortic valve replacement subjects in nonrandomized studies and RCTs.

Three RCTs satisfied modified ROB assessment criteria for “high quality” and three were “low quality” (Additional file 1: Table S4).

### Comparison of treatment effects between RCTs and non-randomized studies

**Fig. 2:**
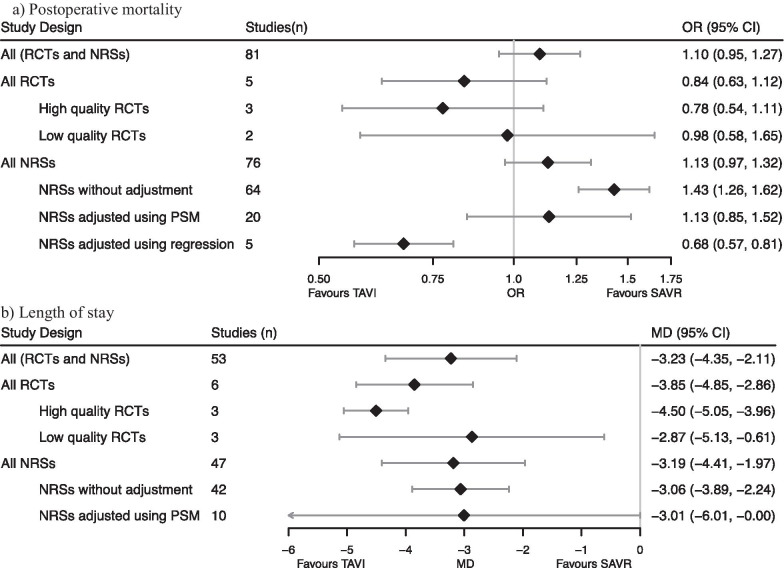
Pooled estimates of treatment effects in different study designs. Abbreviations: RCT, Randomized Controlled Trial; NRS, Non-Randomized Studies; PSM, Propensity Score Matching; OR, Odds Ratio; CI, Confidence Interval; MD, Mean Difference. Early means ≤ 30 days post-op. Diamond is the point estimate of treatment effect. Horizontal lines are 95% CI. Odds ratios were plotted in log scale. Treatment effects were plotted to exact values, but were reported rounded to 2 decimal places

For postoperative mortality, nonrandomized studies adjusted using regression significantly favored transcatheter aortic valve implantation (Fig. 2, OR 0.68 [95% CI 0.57 to 0.81], P for comparison with high quality RCT 0.61). High quality RCTs (OR 0.78 [95% CI 0.54 to 1.11]), low quality RCTs (OR, 0.8 [95% CI 0.58 to 1.65], P for comparison with high quality RCT 0.48) and nonrandomized studies adjusted using PSM (OR, 1.13 [95% CI 0.85 to 1.52], P for comparison with high quality RCT 0.18) found no statistical difference, while nonrandomized studies without adjustment significantly favored surgical aortic valve replacement (OR, 1.43 [95% CI 1.26 to 1.62], P for comparison with high quality RCT 0.01).

For length of stay, all categories of study design except for PSM-adjusted nonrandomized studies significantly favored transcatheter aortic valve implantation (Fig. 2). However, there were differences in the magnitudes of the pooled point estimates. High quality RCTs reported a point estimate for the length of stay in the transcatheter group (MD − 4.50 [95% CI − 5.05 to − 3.96]) that was about 1.5 days shorter than low quality RCTs (MD − 2.87 [95% CI − 5.13 to − 0.61], P for comparison 0.26), nonrandomized studies adjusted using PSM (MD − 3.01 [95% CI − 6.01 to 0], P for comparison 0.62), and nonrandomized studies without adjustment (MD − 3.06 [95% CI − 3.89 to − 2.24], P for comparison 0.33). No nonrandomized studies adjusted length of stay using regression.

### Influence of non-randomized study characteristics on estimates of treatment effect

**Fig. 3:**
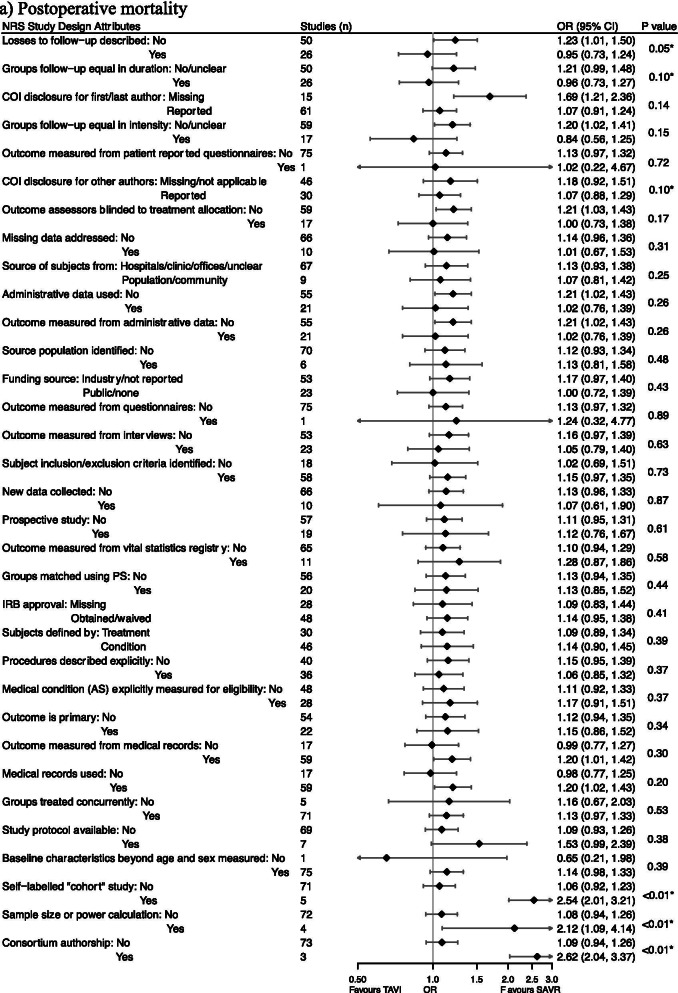

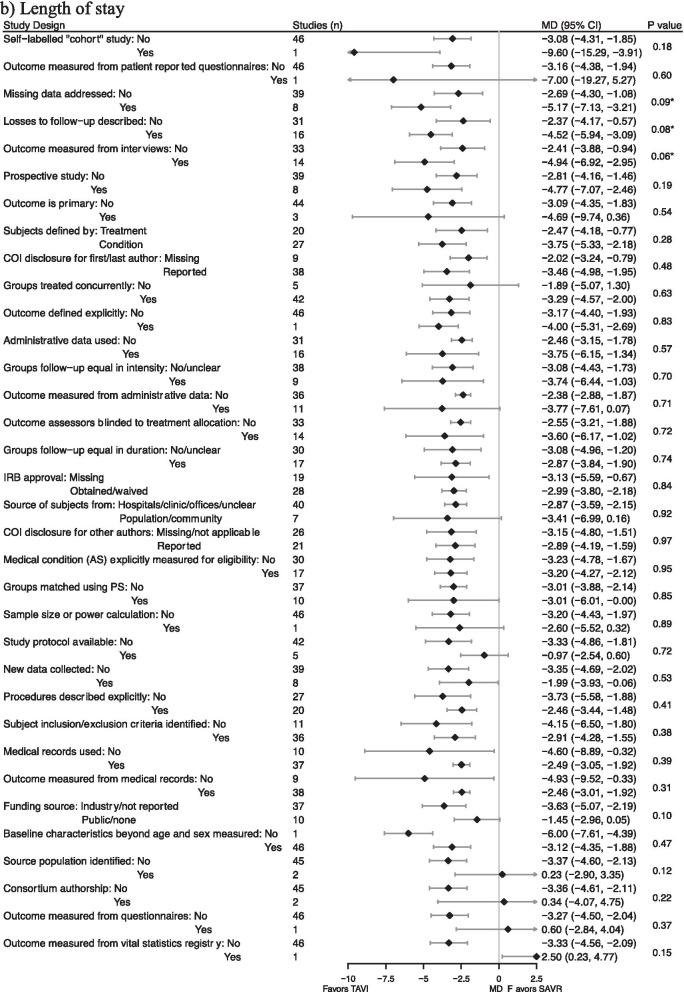
Comparison of pooled estimates of treatment effect in NRSs stratified by specific NRS attributes. Abbreviations: NRS, Non-Randomized Studies; OR, Odds Ratio; MD, Mean Difference; CI, Confidence Interval; COI, Conflict of Interest; IRB, Institutional Review Board; AS, aortic stenosis. Attributes were ordered by increasing Ratio of Odds Ratios (RORs) and Difference in Mean Differences (DMD) between the pooled estimates in each stratification. Diamond is the point estimate. Horizontal lines are 95% CI. Odds ratios were plotted in log scale. Treatment effects are plotted to exact values, but are reported rounded to 2 decimal places. Studies column shows how many studies out of total NRS were pooled to produce each estimate

For each outcome, some attributes of nonrandomized studies were significantly (P < 0.10) associated with pooled estimates of treatment effect closer to those from high quality RCTs (Fig. 3). For postoperative mortality, these attributes were: losses to follow up described (P = 0.05), follow up equal in duration (P = 0.10), and conflict of interest disclosure for non-first/last authors (P = 0.10). For length of stay, these attributes were: losses to follow up described (P = 0.08), missing data addressed (P = 0.09), and outcome measured from interviews (P = 0.06).

## Discussion

When comparing estimates of treatment effects in randomized and nonrandomized studies comparing transcatheter aortic valve implantation with surgical aortic valve replacement, we found that point estimates of the effect sizes of study designs with lower risk of bias tended to show larger benefit for transcatheter aortic valve implantation than study designs with higher risk of bias. Statistical adjustment using regression, but not propensity score matching, brought estimated effect sizes closer to high quality RCTs for postoperative mortality. Among nonrandomized studies, accounting for loss to follow up was associated with estimates of treatment effect closer to those from RCTs.

Our findings are consistent with meta-analyses that found RCTs favored transcatheter aortic valve implantation more than nonrandomized studies with respect to postoperative mortality [31], including a recent meta-analysis analyzing long term (> 2 years) risk of all cause mortality [32]. Interestingly, while meta-epidemiological studies of other clinical topics found that lower quality studies tend to *overestimate* the benefit of newer treatments [19–21, 33–37], higher risk of bias studies of transcatheter aortic valve implantation *underestimated* treatment benefit. There are several possible reasons for the discrepancy. Our analysis included recent studies, which are generally of higher quality and follow better reporting guidelines than older studies [38]. The difference may also be specific to the clinical context we studied. Allocation of patients to treatment groups is highly influenced by differences in case-mix [39]. The surgical risk of postoperative mortality was higher in patients who had transcatheter aortic valve implantation in nonrandomized studies, presumably because the transcatheter procedure was largely restricted to patients who were too high risk for surgical aortic valve replacement in the early years of its clinical use. This situation is different than other clinical situations, where newer or innovative procedures are preferentially used in lower-risk patients [40].

In our study, propensity score matching did not consistently shift estimates from nonrandomized studies closer to RCT estimates, similar to another meta analysis of RCT and propensity score matched nonrandomized studies comparing TAVI with SAVR [41]. Meta analyses in other clinical settings found that propensity score-matched effect sizes from nonrandomized studies were closer to RCTs than regression modeling [39]. The eligibility criteria for inclusion in nonrandomized studies are typically less restrictive than RCTs. The fact that RCTs and nonrandomized studies focus on different groups of patients is an important reason why the results of these studies may differ substantially, and why methods of statistical adjustment can not always rectify the effect of this selection bias.

Of the various design attributes of nonrandomized studies we analyzed, studies that described loss to follow up yielded estimates of treatment effects that were significantly closer to high quality RCTs. Study attributes related to baseline characteristics did not substantially affect effect estimates. Loss to follow up is a major source of selection bias in cohort studies; it is associated with socioeconomic status [42–49], substance abuse [43], smoking [47, 50–53], alcohol abuse [45, 54], physical inactivity [51, 54, 55], and poor diet [54]. Quantifying the extent of bias due to loss to follow up may be helpful in understanding biased estimation of treatment effects in nonrandomized studies.

An important clinical implication of our study is how to interpret nonrandomized studies of new interventions, particularly when patients at higher baseline risk of poor outcomes are preferentially selected for the new intervention. A common pattern in the health literature is that patients at lower baseline risk of poor outcomes are selected for newer and minimally invasive procedures; these patients are often better candidates due to factors such as a smaller burden of disease, or more stable health status. However, in some circumstances, patients at higher risk of poor outcomes might be preferentially treated with the new intervention, especially if the standard treatment is considered very high risk. Clinicians should be very cautious in interpreting the results of nonrandomized comparisons of interventions, and should further be aware that the direction of the bias is unpredictable: it may be expected to favour the new intervention in many cases, but when the standard treatment is a high-risk intervention, the bias may actually favour the standard treatment.

Our study had important strengths. We focused on a single clinical question, allowing us to focus on the influence of study characteristics on estimated treatment effects without introducing other sources of variation from studying a heterogeneous group of interventions. We stratified RCTs by risk of bias, instead of pooling all RCTs together. Studies comparing transcatheter and surgical aortic valve replacement included several high quality RCTs, and many recent large and well-reported nonrandomized studies, enabling us to disentangle the influence of study quality and study characteristics on estimated treatment effects. Thirteen nonrandomized studies reported both adjusted and unadjusted treatment effects. Surgical risk scores allowed us to examine confounding by indication.

Our study has limitations. Our literature review may not have included every potentially eligible study. However, this would not affect the internal consistency and generalizability of our findings, which focused on differential estimates between RCTs and nonrandomized studies, rather than estimating the independent treatment effect of aortic valve replacement techniques. Although we limited our analysis to a single clinical question, there is nevertheless substantial heterogeneity among the articles we analyzed that must be taken into account. Although we categorized studies by design, different studies included subjects from very different clinical populations (e.g., a high-risk population is completely different from an intermediate risk population). However, this situation is typical of the medical literature, and pooled measures of effect are commonly reported in meta-analyses even when clinical heterogeneity exists among included studies. Although some of the high-quality RCTs were designed as non-inferiority studies, they would still be expected to provide unbiased estimates of the relative effectiveness of transcatheter aortic valve implantation with respect to the outcomes we evaluated.

We specified a priori a liberal P value threshold of 0.10 and performed multiple univariate comparisons to identify nonrandomized study attributes potentially associated with biased effect estimates. The intent of these exploratory analyses was to generate hypotheses about these study attributes for future analyses rather than test specific hypotheses. Many of these attributes are correlated, and further research could test specific hypothesis regarding the effect of a limited number of pre-specified attributes on bias. Further studies on the reliability of measured attributes of non-randomized studies and how they influence effect estimates compared with RCTs will help improve the interpretation of the results of nonrandomized studies. Finally, while a single reviewer collected the data for this study, analyses of inter-rater reliability demonstrated excellent correlation among a sample of key variables that were re-abstracted by a second reviewer.

## Conclusion

We found that higher quality studies reported a larger benefit than lower quality studies for transcatheter aortic valve replacement compared with surgical valve replacement, although differences were not statistically significant. While adjusted estimates of treatment effects in nonrandomized studies were generally closer to high quality RCT estimates, propensity score matching and regression modelling varied in the extent to which they were able to adjust effect estimates closer to RCT estimates. Risk adjustment methods may not reliably account for biases in nonrandomized studies. Consideration of loss to follow up appears to be an important attribute of nonrandomized studies with respect to estimating treatment effects that are closer to RCT estimates.

## Supplementary Information

**Additional file 1: Figure S1.** Summary flow chart of research methodology. **Table S1. **Search strategy. **Table S2.** Non-Randomized Study attributes – Definitions, choices, dichotomization, and notes during extraction and analysis. **Table S3.** Citations of included studies. **Table S4. **Cochrane Risk Of Bias (ROB) assessment of RCTs.

## Data Availability

The datasets during and/or analysed during the current study available from the corresponding author on reasonable request.

## Abbreviations

RCT: Randomized controlled trial
PRISMA: Preferred Reporting Items for Systematic Reviews and Meta-Analyses
ICC: Intraclass correlation coefficient
CI: Confidence interval
PSM: Propensity matching score
ROB: Risk of bias
SD: Standard deviation
OR: Odds ratio
MD: Mean difference
ROR: Ratio of odds ratios
DMD: Difference of mean differences
NRS: Nonrandomized Studies
TAVI: Transcatheter aortic valve implantation
SAVR: Surgical aortic valve replacement
