# Supplementary material for: Bias estimation in study design: a meta-epidemiological analysis of transcatheter versus surgical aortic valve replacement
Source: BMC Surg. 2021 Jun 7;21:285. doi: 10.1186/s12893-021-01278-0 (PMC8186071; doi:10.1186/s12893-021-01278-0)
Supplement: Supplementary file 1 — Additional file 1: Figure S1. Summary flow chart of research methodology. Table S1. Search strategy. Table S2. Non-Randomized Study attributes – Definitions, choices, dichotomization, and notes during extraction and analysis. Table S3. Citations of included studies. Table S4. Cochrane Risk Of Bias (ROB) assessment of RCTs. [file 12893_2021_1278_MOESM1_ESM.docx]

**Additional file 1: containing the following online supplementary figures/tables**

**Figure S1.** Summary flow chart of research methodology

**Table S1**. Search strategy

**Table S2**. Non-randomized study attributes: Definition, choices, dichotomization, and notes during extraction and analysis

**Table S3**. Citations of included studies

**Table S4**. Cochrane Risk Of Bias (ROB) assessment of RCTs

**Figure S1.** Summary flow chart of research methodology

RCT, randomized controlled trials; NRS, nonrandomized studies; TAVI, Transcatheter Aortic Valve Implantation; SAVR, Surgical Aortic Valve Replacement

**Table S1.** Search strategy

**Ovid MEDLINE(R) 1946 to June Week 2 2017**

| **#** | **Searches** | **Results** |
| --- | --- | --- |
| 1 | Transcatheter Aortic Valve Replacement/ [ new MeSH as of 2015] | 1729 |
| 2 | TAVI.mp,kw. [ Transcatheter Aortic Valve Implant ] | 2184 |
| 3 | TAVR.mp,kw. [ Transcatheter Aortic Valve Replacement ] | 1042 |
| 4 | (TV-AVI or TVAVI).mp,kw. | 3 |
| 5 | (TA-AVI or TAAVI).mp,kw. | 46 |
| 6 | (tf-AVI or tfAVI).mp,kw. | 7 |
| 7 | ((transcatheter* or percutaneous or transapical* or transfemoral or transcutaneous or transvacular or transarterial) adj2 (aort* or AVR or AVI or replac* or implant* or prosth* or bioprosth* or xenotransplan* or xeno-transplan*)).mp,kw. | 7333 |
| 8 | ((trans-catheter* or percutaneous or trans-apical* or trans-femoral or trans-cutaneous or trans-vacular or trans-arterial) adj2 (aort* or AVR or AVI or replac* or implant* or prosth* or bioprosth* or xenotrans* or xeno-trans*)).mp,kw. | 2111 |
| 9 | or/1-8 [ TAVI or TAVR ] | 7644 |
| 10 | exp aortic valve stenosis/su or aortic stenosis, subvalvular/su or cardiomyopathy, hypertrophic/su or cardiomyopathy, hypertrophic, familial/su or discrete subaortic stenosis/su or aortic stenosis, supravalvular/su or williams syndrome/su | 11023 |
| 11 | (aort* adj3 steno*).mp,kw. and su.fs. [surgery floating subheading ] | 12391 |
| 12 | (aort* and ((surgical or standard or convention* or tradition*) adj3 (AVR or replac* or implant* or prosthe* or bioprosthe* or xenotransplan* or xeno-transplan*))).mp,kw. | 2665 |
| 13 | AVR.mp,kw. [ Aortic Valve Replacement ] | 3613 |
| 14 | SURGICAL AORTIC VALVE REPLACEMENT.mp,kw. [ Surgical Aortic Valve Replacement ] | 302 |
| 15 | SAVI.mp,kw. [ Surgical Aortic Valve Implant ] | 132 |
| 16 | Surgical Aortic Valve Replac*.mp,kw. | 817 |
| 17 | Surgical Aortic Valve Implant*.mp,kw. | 9 |
| 18 | exp Heart Valve Prosthesis/ | 32102 |
| 19 | exp Heart Valve Prosthesis Implantation/ | 19958 |
| 20 | or/10-19 [ SAVI OR SURGICAL AORTIC VALVE REPLACEMENT ] | 53074 |
| 21 | 9 and 20 [ TAVI + SAVI : Comparison Base clinical set ] | 5714 |
| 22 | exp animals/ not (exp animals/ and humans/) | 4416813 |
| 23 | 21 not 22 | 5587 |
| 24 | limit 21 to human | 5572 |
| 25 | 23 or 24 [ limiting to human w/ double-check ] | 5587 |
| 26 | limit 25 to ("all infant (birth to 23 months)" or "all child (0 to 18 years)" or "newborn infant (birth to 1 month)" or "infant (1 to 23 months)" or "preschool child (2 to 5 years)" or "child (6 to 12 years)" or "adolescent (13 to 18 years)") | 113 |
| 27 | 25 not 26 | 5474 |
| 28 | limit 25 to ("all adult (19 plus years)" or "young adult (19 to 24 years)" or "adult (19 to 44 years)" or "young adult and adult (19-24 and 19-44)" or "middle age (45 to 64 years)" or "middle aged (45 plus years)" or "all aged (65 and over)" or "aged (80 and over)") | 3750 |
| 29 | 27 or 28 [ limiting to adult w/ double-check ] | 5535 |
| 30 | exp Clinical Trial/ | 807694 |
| 31 | exp Clinical Trials as Topic/ | 314844 |
| 32 | clinical trial.pt. | 521955 |
| 33 | randomized controlled trial.pt. | 465657 |
| 34 | exp Randomized controlled trial/ | 465835 |
| 35 | exp Randomized Controlled Trials as Topic/ | 116084 |
| 36 | Pragmatic Clinical Trial.pt. | 588 |
| 37 | Pragmatic Clinical Trial/ | 588 |
| 38 | Pragmatic Clinical Trials As Topic/ | 141 |
| 39 | Double-Blind Method/ | 147888 |
| 40 | Random Allocation/ | 93126 |
| 41 | Placebos/ | 35019 |
| 42 | randomi#ed.mp,kw. | 699583 |
| 43 | randomly.mp,kw. | 245765 |
| 44 | (pragmatic adj3 (trial? or study or studies)).mp,kw. | 2707 |
| 45 | placebo*.mp,kw. | 193957 |
| 46 | ((single or double or triple or treble) adj3 (blind* or mask*)).mp,kw. | 204695 |
| 47 | trial.mp,kw. | 1004818 |
| 48 | or/30-47 [ RCTs & related terms ] | 1477191 |
| 49 | 29 and 48 [ (TAVI + SAVI) + RCTs ] | 706 |
| 50 | remove duplicates from 49 [ FINAL "TAVI + SAVI + RCTs" ] | 659 |
| 51 | 29 not 49 [ TAVI + SAVI NOT RCTs ] | 4829 |
| 52 | remove duplicates from 51 [ FINAL "TAVI + SAVI NOT RCTs" ] | 4597 |
| 53 | limit 29 to systematic reviews/ | 264 |
| 54 | (systematic adj4 (review or reviews or overview or overviews)).mp,kw. | 86715 |
| 55 | meta-analysis.pt. | 81378 |
| 56 | meta-analysis as topic/ | 16108 |
| 57 | (meta-anal* or metanal* or metaanal*).mp,kw. | 121475 |
| 58 | metareview*.mp,kw. | 11 |
| 59 | (umbrella adj3 review?).mp,kw. | 71 |
| 60 | (network adj3 review?).mp,kw. | 670 |
| 61 | or/53-60 [ Systematic Reviews & related terms ] | 172550 |
| 62 | 29 and 61 [ (TAVI + SAVI) + SRs or MAs ] | 284 |
| 63 | remove duplicates from 62 | 266 |

**Table S2.** Non-Randomized Study attributes – Definitions, choices, dichotomization, and notes during extraction and analysis

| **Attribute #1**: Consortium authorship | |
| --- | --- |
| **Study or outcome-level**: Study  **Definition**: Is an author part of a consortium? Within the author list, does the study identify “for the X group” or “on behalf of the X collaborators”?  **Notes during extraction/analysis:**   - Often found in citation and/or text   **Present in other NRS assessment tools** (if yes, which?): No | |
| **Choices during:** | |
| **Data extraction** | **Data analysis: Dichotomized versions** |
| Part of consortium | Yes (Better) |
| Not part of consortium | No (Worse) |

| **Attribute #2**: Conflict of interest (COI) disclosure for first/last author | |
| --- | --- |
| **Study or outcome-level**: Study  **Definition**: Identify if conflicts of interest are reported for the first or last author identified in the author list. May be reported as "disclosure(s)" or "competing interests".  **Notes during extraction/analysis:**   - Even if relationship was stated to be unrelated to the study, it was reported as conflict of interest   **Present in other NRS assessment tools** (if yes, which?): No | |
| **Choices during:** | |
| **Data extraction** | **Data analysis: Dichotomized versions** |
| Conflicts of interest reported for either the first or last author | Reported (Better) |
| No conflicts reported by either the first or last author  Missing (conflicts of interest not reported) | Reported (Better)  Missing (Worse) |

| **Attribute #3**: Conflict of interest (COI) disclosure for other authors | |
| --- | --- |
| **Study or outcome-level**: Study  **Definition**: Identify if conflicts of interest are reported by any author who is not the first or last author.  **Notes during extraction/analysis:**   - Even if relationship was stated to be unrelated to the study, it was reported as conflict of interest   **Present in other NRS assessment tools** (if yes, which?): No | |
| **Choices during:** | |
| **Data extraction** | **Data analysis: Dichotomized versions** |
| Conflicts of interest reported for any author who is not the first or last author | Reported (Better) |
| No conflicts reported by any author who is not the first or last author  Missing – conflicts of interest are not reported for any author who is not the first or last author  Not applicable (only 2 authors) | Reported (Better)  Missing (Worse)  Missing (Worse) |

| **Attribute #4**: Source of subjects from | |
| --- | --- |
| **Study or outcome-level**: Study  **Definition**: Identify where the study sample was derived from.  **Notes during extraction/analysis:**  **Present in other NRS assessment tools** (if yes, which?): Yes   - Down and Black Checklist (1998) - Newcastle-Ottawa Scale (2003) | |
| **Choices during:** | |
| **Data extraction** | **Data analysis: Dichotomized versions** |
| Hospital/clinic/office-based, >1 sites | Hospital/clinic/offices/unclear (Worse) |
| Hospital/clinic/office-based, 1 site  Population-based or community-based  Not clear | Hospital/clinic/offices/unclear (Worse)  Population/community (Better)  Hospital/clinic/offices/unclear (Worse) |

| **Attribute #5**: Self-labelled “cohort” study | |
| --- | --- |
| **Study or outcome-level**: Study  **Definition**: What does the study call itself? There should be explicit text phrasing in the study of its type. If the study does not explicitly identify type, chose "study does not identify type".  **Notes during extraction/analysis:**   - Checked for explicit phrasing: “cohort study”   **Present in other NRS assessment tools** (if yes, which?): No | |
| **Choices during:** | |
| **Data extraction** | **Data analysis: Dichotomized versions** |
| Cohort study | Yes (Better) |
| Non-cohort study (i.e.. Case-control, nested case-control)  Study does not identify type | No (Worse)  No (Worse) |

| **Attribute #6**: Assessor-labelled “cohort” study | |
| --- | --- |
| **Study or outcome-level**: study  **Definition**: What would you identify the study as? A nested case-control study is a case-control study performed within a cohort study.  **Notes:**   - Categorized as cohort study if: 1) patients were allocated to TRANSCATHETER AORTIC VALVE IMPLANTATION or SURGICAL AORTIC VALVE REPLACEMENT and 2) were followed over time - Included cohort studies embedded in another study (ex. Cost-effective analysis using primary data)   **Present in other NRS assessment tools** (if yes, which?): No | |
| **Choices during:** | |
| **Data extraction** | **Data analysis: Dichotomized version** |
| Cohort study | Yes (Better) |
| Nested case-control study  Case-control study | No (Worse)  No (Worse) |

| **Attribute #7**: Prospective study | |
| --- | --- |
| **Study or outcome-level**: Study  **Definition**: A **prospective** cohort is a study where subjects are identified prior to the time when outcomes have occurred, and subjects are followed forward in time to determine whether or not an outcome occurs.  A **retrospective** cohort is a study where subjects are identified after both the exposure and outcome have already occurred, even if they have not yet been measured.  There may be explicit text phrasing of the study being ‘prospective’ or ‘retrospective’. For example,  “Both prospective and retrospective data collection” = retrospective chart review was used to identify patients who had surgery and their outcomes after six months and then a (prospective) phone call was placed to assess outcomes at one year;  “Completely prospective data collection” = patients who had surgery were followed for one year, when a clinical assessment was performed;  “Completely retrospective data collection” = charts were reviewed to identify patients treated with a medication and if side effects occurred.  If the study does not explicitly state this, select the most appropriate response for how data was collected AND select the not specified option  **Notes:**   - If not explicitly stated, selected the most appropriate and marked "study does not state" - Did not rely on study calling itself prospective or retrospective, but evaluated whether 1) data was prospectively collected, and 2) study investigators did not have outcome data prior to starting analysis - If study investigators retrospectively analyzed prospectively collected data, considered it retrospective - If study did not report any details about temporal direction of data collection, assumed retrospective, and also selected study does not state - If 1 arm was prospective and 1 arm was retrospective, put both - If 1 outcome was prospective but the rest was retrospective, put both   **Present in other NRS assessment tools** (if yes, which?): Yes   - MINORS (2003) - RTI item bank (2012) - ROBINS-I (2016) | |
| **Choices during:** | |
| **Data extraction** | **Data analysis: Dichotomized version** |
| Both prospective and retrospective data collection | Yes (Better) |
| Completely prospective data collection  Completely retrospective data collection  Study does not state | No (Worse)  No (Worse)  No (Worse) |

| **Attribute #8**: Study protocol available | |
| --- | --- |
| **Study or outcome-level**: Study  **Definition**: A study protocol is a document describing the study procedures that was completed prior to the time that the research activities began. In some cases, protocols are published as stand-alone documents. In other cases, a study may cite a previous publication as the document containing the study protocol. Submitting a protocol for approval by a Research Ethics Board, or evidence of approval by a Research Ethics Board, does not independently satisfy this criterion. Explicit text phrasing of a 'protocol' regarding intervention technique / exposure does not independently satisfy this criterion.  **Notes:**   - Did not count protocol submitted for ethics review, protocol referring to specific procedure or outcome assessment, or mention of protocol without explanation of draft   **Present in other NRS assessment tools** (if yes, which?): No | |
| **Choices during:** | |
| **Data extraction** | **Data analysis: Dichotomized version** |
| Study protocol has been posted, published, or registered; alternately, the study cites a prior study that included the study protocol  Study indicates that a protocol was followed  No study protocol posted, published, registered, or mentioned  Study references a prior publication describing their methods | Yes (Better)  Yes (Better)  No (Worse)  Yes (Better) |

| **Attribute #9**: Institutional Review Board (IRB) approval | |
| --- | --- |
| **Study or outcome-level**: Study  **Definition**: There may be explicit text phrasing of "IRB" ("institutional review board"), "REB" ("research ethics board"), "ethics", "approval", "approved", or "consent" for the study, by "committee(s)".  **Notes:** None  **Present in other NRS assessment tools** (if yes, which?): No | |
| **Choices during:** | |
| **Data extraction** | **Data analysis: Dichotomized version** |
| Ethics board approval or waived requirement  No ethics board approval (study published <2002)  No ethics board approval (study published >2002) | Obtained/waived (Better)  Obtained/waived (Better)  Missing (Worse) |

| **Attribute #10**: New data collected | |
| --- | --- |
| **Study or outcome-level**: Study  **Definition**: New data are collected specifically for the purposes of the study and involve making new measurements from the study participants. If any new data were collected for the study, the response is "Any special information collected for study", even if the study combines the new data with previously collected data. A purely retrospective study has no new data collection and the response would be “No new data collection”. Reviewing charts is not considered new data collection, and the response would be “No new data collection”.  **Notes:**   - Any new data not captured during normal FU considered new (ex. Quality of life) - Char review is not new data - For radiologic data, only considered it new data if imaging is not part of routine check up for patients with aortic valve replacement   **Present in other NRS assessment tools** (if yes, which?): No | |
| **Choices during:** | |
| **Data extraction** | **Data analysis: Dichotomized version** |
| Any special information collected for study (any new data collected)  No new data collection | Yes (Better)  No (Worse) |

| **Attribute #11**: Administrative data used | |
| --- | --- |
| **Study or outcome-level**: Study  **Definition**: Administrative data are collected for routine administrative purposes, such as tracking hospital activity or payment for services, and are not specifically collected for research. Administrative data are almost always held by entities outside the hospital environment, such as government agencies. Large electronic data files containing registry information, such as cancer registries, or vital statistics registries, are also considered administrative data for the purpose of this criterion. The default response is “No administrative data used” if the study does not explicitly mention that administrative data were used.  **Notes:**   - Included population level data with clinical variables that was not collected for research purposes - Did not include any source that is not obviously administrative data (ex. vital statistics, operations/management data), such as registries at any level that houses only clinical or outcomes data, National Inpatient Sample database, surgical databases at single institutions, or "documentation of hospital stay"   **Present in other NRS assessment tools** (if yes, which?): No | |
| **Choices during:** | |
| **Data extraction** | **Data analysis: Dichotomized version** |
| Administrative data used  No administrative data used | Yes (Presence)  No (Absence) |

| **Attribute #12**: Medical records used | |
| --- | --- |
| **Study or outcome-level**: Study  **Definition**: Health records include electronic health records, electronic medical records, or hospital charts, which are routinely collected for the purpose of providing usual patient care.  **Notes:**   - If even one outcome was measured using medical records, chose “electronic health records and/or hospital charts used”, and reflected data source on outcome-level variable too - Includes any institutional databases that are clinical (ex. Department of cardiac surgery database) - If a study was retrospective (explicitly stated or assumed), assumed medical records were used - If a study did not mention any data source, assumed medical records were used - If a study used registries for a single centre or at the population level that is not administrative, assumed medical records were used - If study described sources of outcomes using any of the following terms (ex. Follow up visits, notes from other practitioners, chart, review, record), assumed medical records were used - Did not include studies that have been collected prospectively but analyzed retrospectively by study investigators who know the hypothesis.   **Present in other NRS assessment tools** (if yes, which?): No | |
| **Choices during:** | |
| **Data extraction** | **Data analysis: Dichotomized version** |
| Electronic health records and/or hospital charts used  No electronic health records or hospital charts used | Yes (Presence)  No (Absence) |

| **Attribute #13**: Source population identified | |
| --- | --- |
| **Study or outcome-level**: Study  **Definition**: A source population is a community-based group of people—or population—from which the study subjects were sampled. A source population is generally identified by criteria such as residence in a geographic region (local, regional, or national), and having—or be at risk of developing—the condition of interest.  Without understanding what the source population is, a study cannot appreciate the possible extent of selection bias. Essentially, the source population is the group of persons who would have been included as study subjects had they developed the condition of interest. The source population must not be a convenience sample, or a subset of patients treated in a hospital or clinic, without an explicit indication that the hospital(s) or clinic(s) would capture every case of the condition that occurred among its source population.  *For example,* the following statements describe a source population and would answer “1”: “We mailed surveys to 210,000 nurses in the United States”; “All patients in the Province of Ontario who were newly diagnosed with asthma between January 1, 2003 and December 31, 2009 were included”; “All cases of myocardial infarction occurring in Rochester, Minnesota”; “Our hospital is one of two regional trauma centres providing care to all cases of penetrating trauma in the Greater Toronto Area”. The following example is of study which does not clearly describe a source population: “All patients treated at our hospital for osteoarthritis between January 1, 2003 and December 31, 2009 were included” (unless there is an explicit statement elsewhere that all patients with osteoarthritis in a specified population would be seen at the hospital). “48 Spanish centres” is not identifying source population because the proportion of the Spanish population with disorder is unknown.  **Notes:** None  **Present in other NRS assessment tools** (if yes, which?): No | |
| **Choices during:** | |
| **Data extraction** | **Data analysis: Dichotomized version** |
| Study clearly identifies a source population  Study does not clearly identify a source population | Yes (Better)  No (Worse) |

| **Attribute #14**: Subjects defined by | |
| --- | --- |
| **Study or outcome-level**: Study  **Definition**: Study subjects can be defined by the presence of a medical condition or by the treatment they received. Study subjects defined by the presence of a medical condition are then offered a treatment and outcomes are assessed. For example, the following statement would respond “1”: “Adults with primary achalasia were included in the study and then treated with either pneumatic dilation or laparoscopic Heller myotomy”. The following statement would respond “0”: “Patients who had pneumatic dilation or laparoscopic Heller myotomy were included in the study”.  **Notes:**   - If both outcome and treatment is mentioned, chose condition because we assumed patient sampling began with condition   **Present in other NRS assessment tools** (if yes, which?): No | |
| **Choices during:** | |
| **Data extraction** | **Data analysis: Dichotomized version** |
| Study subjects defined by having a condition  Study subjects defined by having a treatment | Condition (Better)  Treatment (Worse) |

| **Attribute #15**: Subjects inclusion/exclusion criteria identified | |
| --- | --- |
| **Study or outcome-level**: Study  **Definition**: The study should identify one or more eligibility criteria (which consist of inclusion and exclusion criteria) beyond the medical condition of interest.  **Notes:**   - If anything other than condition or treatment was mentioned, including dates, said “yes”   **Present in other NRS assessment tools** (if yes, which?): Yes   - Down and Black (1998) - RTI item bank (2012) - RoBANS (2013) - Q-Coh (2013) | |
| **Choices during:** | |
| **Data extraction** | **Data analysis: Dichotomized version** |
| Inclusion and exclusion criteria are identified  Inclusion and exclusion criteria are not identified | Yes (Better)  No (Worse) |

| **Attribute #16**: Medical condition (Aortic Stenosis; AS) explicitly measured for eligibility | |
| --- | --- |
| **Study or outcome-level**: Study  **Definition**: A criterion is explicit if a person has little or no discretion in determining whether the criterion has been met. To be explicit, a criterion should contain a narrative description that provides specific numerical values or clearly descriptive text. Just listing a correct medical term for a disease, treatment or outcome is not sufficient for “explicit measurement”; some additional information is necessary.  **Notes:**   - If nothing is said about the medical condition (ex. Study subject defined by treatment), then “No”   **Present in other NRS assessment tools** (if yes, which?): No | |
| **Choices during:** | |
| **Data extraction** | **Data analysis: Dichotomized version** |
| Explicit measurement of the eligibility criteria for the medical condition of interest  Not explicit measurement of the eligibility criteria for the medical condition of interest | Yes (Better)  No (Worse) |

| **Attribute #17**: Groups treated concurrently | |
| --- | --- |
| **Study or outcome-level**: Study  **Definition**: Groups may be treated at the same time or one group may have been treated at a distinctly earlier time period (may be referred to as a "historical cohort"). If there is no overlap in the time period when both groups were treated, then groups were treated at different times.  **Notes:**   - If a study describes enrollment as "sequential" from SURGICAL AORTIC VALVE REPLACEMENT to TRANSCATHETER AORTIC VALVE IMPLANTATION, even though there was some overlap, because the default procedures changed over time, said not concurrent   **Present in other NRS assessment tools** (if yes, which?): Yes   - Down and Black (1998) - RTI item bank (2012) - MINORS (2003) - Q-Coh (2013) - ROBINS-I (2016) | |
| **Choices during:** | |
| **Data extraction** | **Data analysis: Dichotomized version** |
| Groups treated during the same general time period  One group was treated at a different time period compared to the other group | Yes (Better)  No (Worse) |

| **Attribute #18**: Groups matched using propensity scores (PS) | |
| --- | --- |
| **Study or outcome-level**: Study  **Definition**: Comparison groups may be matched according to baseline characteristics, generally to reduce the influence of confounding. Matching may be done using a systematic and quantitative approach, such as use of "propensity scores" or random selection to select matched subjects when there are multiple potential matches. In some cases, a study may indicate that some subjects were matched to other subjects in a comparison group, without specifying that a systematic approach was used (for example, “treated patients were matched to untreated patients according to age, sex and smoking status”).  **Notes:**   - Quantitative method means propensity score matching - Nonsystematic matching means matching on specific characteristics without systematic approach - Even if study population is defined by certain characteristic (ex. >90 years old), unless study stated "matched on age", did not count as non systematic/quantitative matching - Matching was considered done only if outcomes of interests were derived from matched cohort - Did not count propensity score adjustment as propensity score matching; only actual selection of group based on propensity score. If both were available (PS score adjusted vs. risk-adjusted PSM cohort), chose the latter to indicate PSM   **Present in other NRS assessment tools** (if yes, which?): Yes   - Down and Black (1998) - Newcastle-Ottawa Scale (2003) - RTI item bank (2012) - MINORS (2003) - ACROVAT-NRSI (2014) - ROBINS-I (2016) | |
| **Choices during:** | |
| **Data extraction** | **Data analysis: Dichotomized version** |
| Matching was done using a systematic or quantitative procedure  Matching was done, but without systematic or quantitative procedure  No matching was done | Yes (Better)  No (Worse)  No (Worse) |

| **Attribute #19**: Baseline characteristics compared | |
| --- | --- |
| **Study or outcome-level**: Study  **Definition**: If baseline characteristics were examined, assessed, or compared, the response is “Baseline characteristics were compared, with explicit values”. This is usually presented as Table 1, with a title explaining the table describes baseline characteristics.  **Notes:** None  **Present in other NRS assessment tools** (if yes, which?): Yes   - Down and Black (1998) | |
| **Choices during:** | |
| **Data extraction** | **Data analysis: Dichotomized version** |
| Baseline characteristics were compared, with explicit values  Study states baseline characteristics were compared but does not provide values  Baseline characteristics were not compared | Quantitatively (Better)  Not compared/compared non-quantitatively (Worse)  Not compared/compared non-quantitatively (Worse) |

| **Attribute #20**: Baseline characteristics beyond age and gender measured | |
| --- | --- |
| **Study or outcome-level**: Study  **Definition**: If any variables beyond basic demographic covariables were measured, such as those relating to the medical condition of interest, the response is “Covariables beyond basic demographics are measured”. Basic demographic covariables include age and gender.  **Notes:**   - If captured anything than age and gender, indicated “yes”   **Present in other NRS assessment tools** (if yes, which?): Yes   - RTI item bank (2012) - RoBANS (2013) - ACROBAT-NRSI (2014) - ROBINS-I (2016) | |
| **Choices during:** | |
| **Data extraction** | **Data analysis: Dichotomized version** |
| Covariables beyond basic demographics are measured  Covariables beyond basic demographics are NOT measured | Yes (Better)  No (Worse) |

| **Attribute #21**: Procedures identified | |
| --- | --- |
| **Study or outcome-level**: Study  **Definition**: Was the intervention / exposure identified in the text?  **Notes:** No  **Present in other NRS assessment tools** (if yes, which?): No | |
| **Choices during:** | |
| **Data extraction** | **Data analysis: Dichotomized version** |
| Interventions(s) were identified  Intervention(s) were NOT identified | Yes (Better)  No (Worse) |

| **Attribute #22**: Procedures described explicitly | |
| --- | --- |
| **Study or outcome-level**: Study  **Definition**: Both interventions under study are described so that other people can understand what the interventions were, and how they were delivered. For example, a drug should be identified by characteristics such as its generic name, dose and route of administration. A surgical procedure should not be defined solely by name (“laparoscopic Heller myotomy with partial fundoplication”; “laparoscopic assisted colectomy”; “Whipple procedure”), but should contain additional information to clearly describe characteristics such as the approach/technique, key steps, extent, operator, or setting.  **Notes:**   - Information that specifically describes how the procedure was done, quantitative description of actual procedure (ex. Size of incision, length of anesthesia, etc.) not statistics on how many people got certain type of valve, etc.   **Present in other NRS assessment tools** (if yes, which?): Yes   - Down and Black (1998) - RTI item bank (2012) - RoBANS (2013) - Q-Coh (2013) - ROBINS-I (2016) | |
| **Choices during:** | |
| **Data extraction** | **Data analysis: Dichotomized version** |
| Explicit description of BOTH interventions with quantitative information  Explicit description of BOTH interventions, without quantitative information  Explicit description of ONLY one of the interventions, with quantitative information  Explicit description of ONLY one of the interventions, without quantitative information  No explicit description of intervention | Yes (Better)  Yes (Better)  No (Worse)  No (Worse)  No (Worse) |

| **Attribute #23**: Study subjects blinded to treatment allocation | |
| --- | --- |
| **Study or outcome-level**: Study  **Definition**: In a study when an assessor determines whether an outcome occurred, the outcome assessors are considered blinded if they were not aware of the study subjects’ treatment allocation at the time that they assessed whether the outcome occurred. If an outcome was self-reported and then confirmed by blinded outcome assessors, the outcome assessors are considered blinded. If assessors were blinded for some outcomes but not others, answer for the primary outcome. In general, studies using administrative data for measuring outcomes are considered blinded.  **Notes:**   - If outcomes of interests were not blinded, even if outcomes that are not outcomes of interest were, said “no” - Administrative data were considered blinded because coders were unaware of study hypothesis   **Present in other NRS assessment tools** (if yes, which?): Yes   - Down and Black (1998) - Newcastle-Ottawa Scale (2003) - MINORS (2003) - RTI item bank (2012) - RoBANS (2013) - Q-Coh (2013) | |
| **Choices during:** | |
| **Data extraction** | **Data analysis: Dichotomized version** |
| Outcome assessors blinded to study subject’s treatment allocation  Outcome assessor not blinded to study subject’s treatment allocation | Yes (Better)  No (Worse) |

| **Attribute #24**: Outcome assessors blinded treatment allocation | |
| --- | --- |
| **Study or outcome-level**: Study  **Definition**: Study subjects were blinded if they were not aware of their treatment allocation during the study and follow-up.  **Notes:** None  **Present in other NRS assessment tools** (if yes, which?): Yes   - Down and Black (1998) - Q-Coh (2013) | |
| **Choices during:** | |
| **Data extraction** | **Data analysis: Dichotomized version** |
| Study subjects blinded  Study subjects NOT blinded | Yes (Better)  No (Worse) |

| **Attribute #25**: Sample size or power calculation estimated | |
| --- | --- |
| **Study or outcome-level**: Study  **Definition**: Sample size calculation done.  **Notes:**   - Need quantitative estimates. If described why sample size is good enough, said “No”   **Present in other NRS assessment tools** (if yes, which?): Yes   - Down and Black (1998) - MINORS (2003) - RTI item bank (2012) | |
| **Choices during:** | |
| **Data extraction** | **Data analysis: Dichotomized version** |
| Sample size calculation was described, or in the absence of a sample size calculation, a quantitative estimate of statistical power was provided  No sample size calculation was described no statistical power estimate was provided | Yes (Better)  No (Worse) |

| **Attribute #26**: Groups follow-up equal in duration | |
| --- | --- |
| **Study or outcome-level**: Study  **Definition**: Was follow-up equivalent in both groups, as stated by the study? For example, if one treatment group was followed for 1 year while the other group was followed for 1 month, the response would be “Follow-up of higher intensity in one group, follow-up of lesser intensity in other group”. If the difference is more than 50% follow-up is unequal.  **Notes:**   - Unless stated completely clearly (duration of follow up stated for both groups), said “unclear” - If TRANSCATHETER AORTIC VALVE IMPLANTATION had more than 1 arm divided by any baseline characteristic (ex. Approach, surgical risk), and arms have different follow up schedules, said “unclear”   **Present in other NRS assessment tools** (if yes, which?): Yes   - Down and Black (1998) - RTI item bank (2012) - Q-Coh (2013) | |
| **Choices during:** | |
| **Data extraction** | **Data analysis: Dichotomized version** |
| Follow-up equal duration in both groups  Follow-up shorter in one group  Unclear | Yes (Better)  No/unclear (Worse)  No/unclear (Worse) |

| **Attribute #27**: Groups follow-up equal in intensity | |
| --- | --- |
| **Study or outcome-level**: Study  **Definition**: Was the schedule/format of outcome assessment similar in both groups, as stated by the study, or was one treatment group followed more rigorously than the other? For example, if there is the same periodicity and approach in both groups, the response would be “follow-up of equal intensity in both groups”. If one group was monitored daily for the occurrence of an outcome, but the other group was monitored weekly, the response would be “study does not describe losses to follow-up”.  **Notes:**   - Unless stated completely clearly, said unclear - If stated in any way some consistent follow up schedule for any outcome (“measured at x time points”), said “follow-up of equal intensity in both groups” because assumed other outcomes were checked up at same time points. Did not count outcomes being reported a specific time point (“x at discharge”, “x at 1-year”), but sentence specifically referring to follow up schedule - If said follow up was "recommended" at specific time points, no indication it did happen, so said “unclear” - If discharged to different places, assumed “unclear” since follow up schedules can differ   **Present in other NRS assessment tools** (if yes, which?): No | |
| **Choices during:** | |
| **Data extraction** | **Data analysis: Dichotomized version** |
| Follow-up of equal intensity in both groups  Follow-up of higher intensity in one group, follow-up of lesser intensity in other group  Unclear | Yes (Better)  No/unclear (Worse)  No/unclear (Worse) |

| **Attribute #28**: Losses to follow-up described | |
| --- | --- |
| **Study or outcome-level**: Study  **Definition**: Loss to follow up is described in some manner, such as describing the proportion of subjects for whom outcome information is available, or how many subjects dropped out of the study  **Notes:**   - Search for texts “loss”, “lost”, “follow up”   **Present in other NRS assessment tools** (if yes, which?): Yes   - Down and Black (1998) - Newcastle-Ottawa Scale (2003) - MINORS (2003) - RTI item bank (2012) | |
| **Choices during:** | |
| **Data extraction** | **Data analysis: Dichotomized version** |
| Study describes losses to follow-up  Study does not describe losses to follow-up | Yes (Better)  No/unclear (Worse) |

| **Attribute #29**: Missing data addressed | |
| --- | --- |
| **Study or outcome-level**: Study  **Definition**: The impact of missing data may be explored or managed in various ways, such as imputation techniques, or sensitivity analyses estimating the outcome among groups with no missing data.  **Notes:**   - Even if no missing data was addressed for a segment of follow up, because it was not addressed for the entirety of follow-up, said no because it is study level   **Present in other NRS assessment tools** (if yes, which?): Yes   - RoBANS (2013) - ACROBTA-NRSI (2014) - ROBINS-I (2016) | |
| **Choices during:** | |
| **Data extraction** | **Data analysis: Dichotomized version** |
| Imputation techniques or sensitivity analyses presented, or statement that no data were missing  Handling of missing data not addressed, or subjects with missing data simply excluded | Yes (Better)  No (Worse) |

| **Attribute #30**: Funding source | |
| --- | --- |
| **Study or outcome-level**: Study  **Definition**: Financial support can be from industry or non-industry. Non-financial support can include the provision of study supplies (eg, drugs), which may be from industry or non-industry.  **Notes:** None  **Present in other NRS assessment tools** (if yes, which?): Yes   - RTI item bank (2012) | |
| **Choices during:** | |
| **Data extraction** | **Data analysis: Dichotomized version** |
| Any industry, manufacturer, or vendor provides any financial or non-financial support  Non-corporate (public, government agency, granting agency) or private (non-profit) foundation provides financial or non-financial support  No sources of financial or non-financial support  Not reported | Industry/not reported (Worse)  Public/none (Better)  Public/none (Better)  Industry/not reported (Worse) |

Outcome-level attributes (#31-41)

| **Attribute #31**: Outcome is primary | |
| --- | --- |
| **Study or outcome-level**: Outcome  **Definition**: A primary outcome is explicitly identified in the article using the exact phrase “primary outcome”, or can be inferred if all other outcomes except for a single main outcome of interest are labelled “secondary outcomes”.  **Notes:** None  **Present in other NRS assessment tools** (if yes, which?): No | |
| **Choices during:** | |
| **Data extraction** | **Data analysis: Dichotomized version** |
| Outcome is explicitly identified by the study as a primary outcome  Outcome is not explicitly identified as a primary outcome, but can be inferred to be (all other outcomes are referred to as secondary/additional outcomes)  Outcome is not the study's primary outcome | Yes (Presence)  No (Worse)  No (Worse) |

| **Attribute #32**: Outcome measured from vital statistics registry | |
| --- | --- |
| **Study or outcome-level**: Outcome  **Definition**: How was this outcome measured? Determine the source of outcome was derived from.  **Notes:**   - Considered national health and wellness databases as both vital statistics registry and administrative database - If outcome was from administrative data or health record, was reflected on study-level attribute   **Present in other NRS assessment tools** (if yes, which?): Yes   - RTI item bank (2012) | |
| **Choices during:** | |
| **Data extraction** | **Data analysis: Dichotomized version** |
| Vital statistics registry  All other sources (administrative data, physiological measurement, health record, interview, patient reported questionnaire, questionnaire) | Yes (Presence)  No (Absence) |

| **Attribute #33**: Outcome measured from administrative data | |
| --- | --- |
| **Study or outcome-level**: Outcome  **Definition**: How was this outcome measured? Determine the source of outcome was derived from.  **Notes:**   - Considered national health and wellness databases as both vital statistics registry and administrative database - If outcome was from administrative data or health record, was reflected on study-level attribute   **Present in other NRS assessment tools** (if yes, which?): Yes   - RTI item bank (2012) | |
| **Choices during:** | |
| **Data extraction** | **Data analysis: Dichotomized version** |
| Administrative data  All other sources (Vital statistics registry, physiological measurement, health record, interview, patient reported questionnaire, questionnaire) | Yes (Presence)  No (Absence) |

| **Attribute #34**: Outcome measured from physiological measurement | |
| --- | --- |
| **Study or outcome-level**: Outcome  **Definition**: How was this outcome measured? Determine the source of outcome was derived from.  **Notes:**   - Assumed all-cause mortality is a physiological measurement. Assumed LENGTH OF POSTPROCEDURAL STAY was NOT a physiological measurement   **Present in other NRS assessment tools** (if yes, which?): Yes   - RTI item bank (2012) | |
| **Choices during:** | |
| **Data extraction** | **Data analysis: Dichotomized version** |
| Physiological measurement (e.g., clinical findings, laboratory tests, imaging findings, pathologic findings)  All other sources (Vital statistics registry, administrative data, health record, interview, patient reported questionnaire, questionnaire) | Yes (Presence)  No (Absence) |

| **Attribute #35**: Outcome measured from medical records | |
| --- | --- |
| **Study or outcome-level**: Outcome  **Definition**: How was this outcome measured? Determine the source of outcome was derived from.  **Notes:**   - If assumed at study level data source used was health records, reflected on outcome   **Present in other NRS assessment tools** (if yes, which?): Yes   - RTI item bank (2012) | |
| **Choices during:** | |
| **Data extraction** | **Data analysis: Dichotomized version** |
| Health record  All other sources (Vital statistics registry, administrative data, physiological measurement, interview, patient reported questionnaire, questionnaire) | Yes (Presence)  No (Absence) |

| **Attribute #36**: Outcome measured from interviews | |
| --- | --- |
| **Study or outcome-level**: Outcome  **Definition**: How was this outcome measured? Determine the source of outcome was derived from.  **Notes:**   - Telephone contact was considered to be “interview”   **Present in other NRS assessment tools** (if yes, which?): Yes   - RTI item bank (2012) | |
| **Choices during:** | |
| **Data extraction** | **Data analysis: Dichotomized version** |
| Interview  All other sources (Vital statistics registry, administrative data, physiological measurement, health record, patient reported questionnaire, questionnaire) | Yes (Presence)  No (Absence) |

| **Attribute #37**: Outcome measured from patient reported questionnaires | |
| --- | --- |
| **Study or outcome-level**: Outcome  **Definition**: How was this outcome measured? Determine the source of outcome was derived from.  **Notes:** None  **Present in other NRS assessment tools** (if yes, which?): Yes   - RTI item bank (2012) | |
| **Choices during:** | |
| **Data extraction** | **Data analysis: Dichotomized version** |
| Patient reported questionnaire  All other sources (Vital statistics registry, administrative data, physiological measurement, health record, interview, questionnaire) | Yes (Presence)  No (Absence) |

| **Attribute #38**: Outcome measured from questionnaires | |
| --- | --- |
| **Study or outcome-level**: Outcome  **Definition**: How was this outcome measured? Determine the source of outcome was derived from.  **Notes:** None  **Present in other NRS assessment tools** (if yes, which?): Yes   - RTI item bank (2012) | |
| **Choices during:** | |
| **Data extraction** | **Data analysis: Dichotomized version** |
| Questionnaire  All other sources (Vital statistics registry, administrative data, physiological measurement, health record, interview, patient reported questionnaire) | Yes (Presence)  No (Absence) |

| **Attribute #39**: Outcome defined explicitly | |
| --- | --- |
| **Study or outcome-level**: Outcome  **Definition**: An outcome definition is explicit if a person has little or no discretion in determining whether the criteria for the occurrence of the outcome have been met. To be explicit, a criterion should contain a description that provides specific numerical values or a pathological diagnosis. For administrative data, listing of specific codes is explicit. Just listing a correct medical term for a disease, treatment or outcome is not sufficient. For example, “treatment failure” or “need for re-treatment” are not explicit outcomes if specific quantitative criteria and a threshold for these concepts are not explicitly provided. Death is generally considered an explicit outcome.  **Notes:**   - ICD codes were assumed to be explicit - All-cause mortality was assumed to be explicit - Length of stay was assumed to be explicit only if specific dates that was used to calculate it was described (date of operation, date discharged, etc.)   **Present in other NRS assessment tools** (if yes, which?): Yes   - Q-Coh (2013) | |
| **Choices during:** | |
| **Data extraction** | **Data analysis: Dichotomized version** |
| Criterion contains a description that provides specific numerical values / codes or a pathological diagnosis  Criterion does not contain a description that provides specific numerical values / codes or a pathological diagnosis | Yes (Better)  No (Worse) |

| **Attribute #40**: Outcome objective | |
| --- | --- |
| **Study or outcome-level**: Outcome  **Definition**: An objective outcome is an outcome defined by vital signs, radiologic findings, pathologic findings, laboratory values, or physiologic measurements. For administrative data, an outcome is objective if the way in which it was measured is clearly described in the article as being defined by measurement of vital signs, radiologic findings, pathologic findings, laboratory values, or physiologic measurements. In most instances, outcomes measured in administrative data sets are not objective. Cancer is an objective outcome if measured by a cancer registry that requires pathologic confirmation (diagnosis of cancer is usually based on pathologic findings).  **Notes:**   - If outcome was derived from admin data, it was considered to be not objective. ICD codes were not objective because assessor cannot be sure on how they were measured - Death is a physiological measurement and therefor is objective - Length of stay is considered objective because there can be no ambiguity about whether the patient stayed in hospital or not   **Present in other NRS assessment tools** (if yes, which?): No | |
| **Choices during:** | |
| **Data extraction** | **Data analysis: Dichotomized version** |
| Objective outcome  Not objective outcome | Yes (Better)  No (Worse) |

| **Attribute #42**: Outcome assessed at dates | |
| --- | --- |
| **Study or outcome-level**: Outcome  **Definition**: When was the outcome assessed? If assessed at relative duration from date treated, date will be consistent across studies. If assessed at fixed time regardless of date treated, date will not be consistent across studies.  **Notes:**   - Assumed all-cause mortality was measured at date relative from date treated because studies always specified at which time point postop it was measured - Assumed length of stay post-op was measured at relative date because we assumed patients were seen when they were discharged, and date of discharge would be recorded   **Present in other NRS assessment tools** (if yes, which?): Yes   - Q-Coh (2013) | |
| **Choices during:** | |
| **Data extraction** | **Data analysis: Dichotomized version** |
| At a specific time after exposure (eg, 1 year after treatment) or a time-to-event, where all patients could have been followed for the same amount of time  At a specific date, not at a specified time after exposure | Relative duration from date treated (Better)  Fixed duration from date treated (Worse) |

**Table S3**. Citations of included studies

|  | RCT (n=6) |
| --- | --- |
|  | **D. H. Adams, J. J. Popma, M. J. Reardon, S. J. Yakubov, J. S. Coselli, M. D. Deeb, T. G. Gleason, M. Buchbinder, J. Hermiller Jr., N. S. Kleiman, S. Chetcuti, J. Heiser, W. Merhi, G. Zorn, P. Tadros, N. Robinson, G. Petrossian, C. Hughes, K. Harrison, J. Conte, B. Maini, M. Mumtaz, S. Chenoweth, M. S., J. K. Oh, U. S. Clinical Investigators CoreValve.** Transcatheter Aortic-Valve Replacement with a Self-Expanding Prosthesis. *New England Journal of Medicine.* 2014. 370:1790-8 |
|  | **H. G. Thyregod,​ D. A. Steinbruchel,​ N. Ihlemann,​ H. Nissen,​ B. J. Kjeldsen,​ P. Petursson,​ Y. Chang,​ O. W. Franzen,​ T. Engstrom,​ P. Clemmensen,​ P. B. Hansen,​ L. W. Andersen,​ P. S. Olsen,​ L. Sondergaard**. Transcatheter Versus Surgical Aortic Valve Replacement in Patients With Severe Aortic Valve Stenosis: 1-Year Results From the All-Comers NOTION Randomized Clinical Trial. *Journal of the American College of Cardiology.* 2015. 65:2184-94 |
|  | **H. H. M. Nielsen,​ K. E. Klaaborg,​ H. Nissen,​ K. Terp,​ P. E. Mortensen,​ B. J. Kjeldsen,​ C. J. Jakobsen,​ H. R. Andersen,​ H. Egeblad,​ L. R. Krusell,​ L. Thuesen,​ V. E. Hjortdal**. A prospective,​ randomised trial of transapical transcatheter aortic valve implantation vs. surgical aortic valve replacement in operable elderly patients with aortic stenosis: The STACCATO trial. *EuroIntervention.* 2012. 8:383-389 |
|  | **M. B. Leon,​ C. R. Smith,​ M. J. Mack,​ R. R. Makkar,​ L. G. Svensson,​ S. K. Kodali,​ V. H. Thourani,​ E. M. Tuzcu,​ D. C. Miller,​ H. C. Herrmann,​ D. Doshi,​ D. J. Cohen,​ A. D. Pichard,​ S. Kapadia,​ T. Dewey,​ V. Babaliaros,​ W. Y. Szeto,​ M. R. Williams,​ D. Kereiakes,​ A. Zajarias,​ K. L. Greason,​ B. K. Whisenant,​ R. W. Hodson,​ J. W. Moses,​ A. Trento,​ D. L. Brown,​ W. F. Fearon,​ P. Pibarot,​ R. T. Hahn,​ W. A. Jaber,​ W. N. Anderson,​ M. C. Alu,​ J. G. Webb,​ Partner Investigators**. Transcatheter or Surgical Aortic-Valve Replacement in Intermediate-Risk Patients. *New England Journal of Medicine.* 2016. 374:1609-20 |
|  | **C. R. Smith, M. B. Leon, M. J. Mack, C. Miller, J. W. Moses, L. G. Svensson, M. Tuzcu, J. G. Webb, G. P. Fontana, R. R. Makkar, M. Williams, T. Dewey, S. Kapadia, V. Babaliaros, V. H. Thourani, P. Corso, A. D. Pichard, J. E. Bavaria, H. C. Herrmann, J. J. Akin, W. N. Anderson, D. Wang, S. J. Pocock, Partner trial investigators.** Transcatheter versus Surgical Aortic Valve Replacement in High-Risk Patients. *New England Journal of Medicine.* 2011. 364:2187-98 |
|  | **M. J. Reardon,​ N. M. Van Mieghem,​ J. J. Popma,​ N. S. Kleiman,​ L. Sondergaard,​ M. Mumtaz,​ D. H. Adams,​ G. M. Deeb,​ B. Maini,​ H. Gada,​ S. Chetcuti,​ T. Gleason,​ J. Heiser,​ R. Lange,​ W. Merhi,​ J. K. Oh,​ P. S. Olsen,​ N. Piazza,​ M. Williams,​ S. Windecker,​ S. J. Yakubov,​ E. Grube,​ R. Makkar,​ J. S. Lee,​ J. Conte,​ E. Vang,​ H. Nguyen,​ Y. Chang,​ A. S. Mugglin,​ P. W. Serruys,​ A. P. Kappetein,​ Surtavi Investigators**. Surgical or Transcatheter Aortic-Valve Replacement in Intermediate-Risk Patients. *New England Journal of Medicine.* 2017. 376:1321-1331 |

Referencing the earliest publication from each trial.

|  | NRS (n=87) |
| --- | --- |
|  | **A. Alassar,​ G. Soppa,​ M. Edsell,​ P. Rich,​ D. Roy,​ I. Chis Ster,​ R. Joyce,​ O. Valencia,​ T. Barrick,​ F. Howe,​ N. Moat,​ R. Morris,​ H. S. Markus,​ M. Jahangiri**. Incidence and mechanisms of cerebral ischemia after transcatheter aortic valve implantation compared with surgical aortic valve replacement. *Annals of Thoracic Surgery.* 2015. 99:802-8 |
|  | **A. Blehm,​ V. A. Sorokin,​ M. Hartman,​ K. L. Wai,​ K. Schmitz,​ A. Lichtenberg**. Quality of Life Shift after Aortic Valve Replacement in the Era of TAVI: Single-Center Class Comparison Study Between Different Procedural Techniques. *Journal of Heart Valve Disease.* 2015. 24:540-53 |
|  | **A. D'Onofrio,​ G. Rizzoli,​ A. Messina,​ O. Alfieri,​ R. Lorusso,​ S. Salizzoni,​ M. Glauber,​ R. Di Bartolomeo,​ L. Besola,​ M. Rinaldi,​ G. Troise,​ G. Gerosa**. Conventional surgery,​ sutureless valves,​ and transapical aortic valve replacement: what is the best option for patients with aortic valve stenosis? A multicenter,​ propensity-matched analysis. *Journal of Thoracic & Cardiovascular Surgery.* 2013. 146:1065-70; discussion 1070-1 |
|  | **A. D'Onofrio,​ O. R. Alfieri,​ M. Cioni,​ F. Alamanni,​ M. Fusari,​ V. Tarzia,​ G. Rizzoli,​ G. Gerosa**. The impact of transcatheter aortic valve implantation on patients' profiles and outcomes of aortic valve surgery programmes: a multi-institutional appraisal. *Interactive Cardiovascular & Thoracic Surgery.* 2013. 16:608-11 |
|  | **A. Latib,​ F. Maisano,​ L. Bertoldi,​ A. Giacomini,​ J. Shannon,​ M. Cioni,​ A. Ielasi,​ F. Figini,​ K. Tagaki,​ A. Franco,​ R. D. Covello,​ A. Grimaldi,​ P. Spagnolo,​ G. L. Buchannan,​ M. Carlino,​ A. Chieffo,​ M. Montorfano,​ O. Alfieri,​ A. Colombo**. Transcatheter vs surgical aortic valve replacement in intermediate-surgical-risk patients with aortic stenosis: a propensity score-matched case-control study. *American Heart Journal.* 2012. 164:910-7 |
|  | **A. Ribera,​ J. Slof,​ R. Andrea,​ C. Falces,​ E. Gutierrez,​ R. Del Valle-Fernandez,​ C. Moris-de la Tassa,​ P. Mota,​ J. F. Oteo,​ P. Cascant,​ O. A. Altisent,​ C. Sureda,​ V. Serra,​ B. Garcia-Del Blanco,​ P. Tornos,​ D. Garcia-Dorado,​ I. Ferreira-Gonzalez**. Transfemoral transcatheter aortic valve replacement compared with surgical replacement in patients with severe aortic stenosis and comparable risk: cost-utility and its determinants. *International Journal of Cardiology.* 2015. 182:321-8 |
|  | **Anja Gutmann,​ Klaus Kaier,​ Holger Reinecke,​ Lutz Frankenstein,​ Andreas Zirlik,​ Wolfgang Bothe,​ Constantin von Zur Muhlen,​ Manfred Zehender,​ Jochen Reinohl,​ Christoph Bode,​ Peter Stachon**. Impact of pulmonary hypertension on in-hospital outcome after surgical or transcatheter aortic valve replacement. *Eurointervention.* 2017. 25:25 |
|  | **B. E. Stahli,​ J. Grunenfelder,​ S. Jacobs,​ V. Falk,​ U. Landmesser,​ M. B. Wischnewsky,​ T. F. Luscher,​ R. Corti,​ W. Maier,​ L. A. Altwegg**. Assessment of inflammatory response to transfemoral transcatheter aortic valve implantation compared to transapical and surgical procedures: a pilot study. *Journal of Invasive Cardiology.* 2012. 24:407-11 |
|  | **B. Retzlaff,​ N. Wessel,​ M. Riedl,​ A. Gapelyuk,​ H. Malberg,​ N. Bauernschmitt,​ J. Kurths,​ G. Bretthauer,​ R. Bauernschmitt**. Preserved autonomic regulation in patients undergoing transcatheter aortic valve implantation (TAVI): a prospective,​ comparative study. *Biomedizinische Technik.* 2011. 56:185-93 |
|  | **B. Thakkar,​ A. Patel,​ B. Mohamad,​ N. J. Patel,​ P. Bhatt,​ R. Bhimani,​ A. Patel,​ S. Arora,​ C. Savani,​ S. Solanki,​ R. Sonani,​ S. Patel,​ N. Patel,​ A. Deshmukh,​ T. Mohamad,​ C. Grines,​ M. Cleman,​ A. Mangi,​ J. Forrest,​ A. O. Badheka**. Transcatheter aortic valve replacement versus surgical aortic valve replacement in patients with cirrhosis. *Catheterization & Cardiovascular Interventions.* 2016. 87:955-62 |
|  | **Beatriz Vaquerizo,​ Sabine Bleiziffer,​ Michael Wottke,​ Marco Spaziano,​ Lena Eschenbach,​ Rudiger Lange,​ Nicolo Piazza**. Impact of transcatheter aortic valve implantation on surgical aortic valve. *International Journal of Cardiology.* 2017. 19:19 |
|  | **C. Adrie,​ M. Parlato,​ L. Salmi,​ M. Adib-Conquy,​ O. Bical,​ P. Deleuze,​ C. Fitting,​ J. M. Cavaillon,​ M. Monchi**. Bacterial translocation and plasma cytokines during transcatheter and open-heart aortic valve implantation. *Shock.* 2015. 43:62-67 |
|  | **C. Dubois,​ M. Coosemans,​ F. Rega,​ G. Poortmans,​ A. Belmans,​ T. Adriaenssens,​ M. C. Herregods,​ K. Goetschalckx,​ W. Desmet,​ S. Janssens,​ B. Meyns,​ P. Herijgers**. Prospective evaluation of clinical outcomes in all-comer high-risk patients with aortic valve stenosis undergoing medical treatment,​ transcatheter or surgical aortic valve implantation following heart team assessment. *Interactive Cardiovascular & Thoracic Surgery.* 2013. 17:492-500 |
|  | **C. F. Appel,​ H. Hultkvist,​ E. Nylander,​ H. Ahn,​ N. E. Nielsen,​ W. Freter,​ F. Vanky**. Transcatheter versus surgical treatment for aortic stenosis: patient selection and early outcome. *Scandinavian Cardiovascular Journal.* 2012. 46:301-7 |
|  | **C. Gavina,​ A. Goncalves,​ C. Almeria,​ R. Hernandez,​ A. Leite-Moreira,​ F. Rocha-Goncalves,​ J. Zamorano**. Determinants of clinical improvement after surgical replacement or transcatheter aortic valve implantation for isolated aortic stenosis. *Cardiovascular Ultrasound.* 2014. 12 (1) (no pagination):#pages# |
|  | **C. J. O'Sullivan,​ L. Englberger,​ N. Hosek,​ D. Heg,​ D. Cao,​ G. G. Stefanini,​ S. Stortecky,​ S. Gloekler,​ E. Spitzer,​ D. Tuller,​ C. Huber,​ T. Pilgrim,​ F. Praz,​ L. Buellesfeld,​ A. A. Khattab,​ T. Carrel,​ B. Meier,​ S. Windecker,​ P. Wenaweser**. Clinical outcomes and revascularization strategies in patients with low-flow,​ low-gradient severe aortic valve stenosis according to the assigned treatment modality. *Jacc: Cardiovascular Interventions.* 2015. 8:704-17 |
|  | **C. J. Zack,​ F. Al-Qahtani,​ A. Kawsara,​ M. Al-Hijji,​ A. H. Amin,​ M. Alkhouli**. Comparative Outcomes of Surgical and Transcatheter Aortic Valve Replacement for Aortic Stenosis in Nonagenarians. *American Journal of Cardiology.* 2017. 119:893-899 |
|  | **C. Kocaaslan,​ B. Ketenci,​ M. Yilmaz,​ T. Kehlibar,​ M. E. Memetoglu,​ G. Ertas,​ M. Eren,​ M. M. Demirtas**. Comparison of Transcatheter Aortic Valve Implantation versus Surgical Aortic Valve Replacement to Improve Quality of Life in Patients >70 Years of Age with Severe Aortic Stenosis. *Brazilian Journal of Cardiovascular Surgery.* 2016. 31:1-6 |
|  | **C. Muneretto,​ G. Bisleri,​ A. Moggi,​ L. Di Bacco,​ M. Tespili,​ A. Repossini,​ M. Rambaldini**. Treating the patients in the 'grey-zone' with aortic valve disease: a comparison among conventional surgery,​ sutureless valves and transcatheter aortic valve replacement. *Interactive Cardiovascular & Thoracic Surgery.* 2015. 20:90-5 |
|  | **C. Muneretto,​ O. Alfieri,​ B. M. Cesana,​ G. Bisleri,​ M. De Bonis,​ R. Di Bartolomeo,​ C. Savini,​ G. Folesani,​ L. Di Bacco,​ M. Rambaldini,​ J. P. Maureira,​ F. Laborde,​ M. Tespili,​ A. Repossini,​ T. Folliguet**. A comparison of conventional surgery,​ transcatheter aortic valve replacement,​ and sutureless valves in "real-world" patients with aortic stenosis and intermediate- to high-risk profile. *Journal of Thoracic & Cardiovascular Surgery.* 2015. 150:1570-7; discussion 1577-9 |
|  | **C. Tamburino,​ M. Barbanti,​ D. Capodanno,​ C. Mignosa,​ M. Gentile,​ P. Aruta,​ A. M. Pistritto,​ C. Bonanno,​ S. Bonura,​ A. Cadoni,​ S. Gulino,​ M. C. Di Pasqua,​ V. Cammalleri,​ M. Scarabelli,​ M. Mule,​ S. Imme,​ G. Del Campo,​ G. P. Ussia**. Comparison of complications and outcomes to one year of transcatheter aortic valve implantation versus surgical aortic valve replacement in patients with severe aortic stenosis. *American Journal of Cardiology.* 2012. 109:1487-93 |
|  | **Carlos Rodriguez-Pascual,​ Emilio Paredes-Galan,​ Ana Isabel Ferrero-Martinez,​ Jose Antonio Baz-Alonso,​ Dario Duran-Munoz,​ Eva Gonzalez-Babarro,​ Marcelo Sanmartin,​ Teresa Parajes,​ Ivett Torres-Torres,​ Miguel Pinon-Esteban,​ Francisco Calvo-Iglesias,​ Maria Teresa Olcoz-Chiva,​ Fernando Rodriguez-Artalejo**. The frailty syndrome and mortality among very old patients with symptomatic severe aortic stenosis under different treatments. *International Journal of Cardiology.* 2016. 224:125-131 |
|  | **Claire Bouleti,​ Marion Chauvet,​ Guillaume Franchineau,​ Dominique Himbert,​ Bernard Iung,​ Benjamin Alos,​ Eric Brochet,​ Marina Urena,​ Walid Ghodbane,​ Phalla Ou,​ Sophie Provenchere,​ Patrick Nataf,​ Alec Vahanian**. The impact of the development of transcatheter aortic valve implantation on the management of severe aortic stenosis in high-risk patients: treatment strategies and outcome. *European Journal of Cardio-Thoracic Surgery.* 2017. 51:80-88 |
|  | **D. Dvir,​ A. Sagie,​ E. Porat,​ A. Assali,​ Y. Shapira,​ H. Vaknin-Assa,​ G. Shafir,​ T. Bental,​ R. Nevzorov,​ A. Battler,​ R. Kornowski**. Clinical profile and outcome of patients with severe aortic stenosis at high surgical risk: single-center prospective evaluation according to treatment assignment. *Catheterization & Cardiovascular Interventions.* 2013. 81:871-81 |
|  | **D. M. Kobrin,​ F. H. McCarthy,​ H. C. Herrmann,​ S. Anwaruddin,​ S. Kobrin,​ W. Y. Szeto,​ J. E. Bavaria,​ P. W. Groeneveld,​ N. D. Desai**. Transcatheter and Surgical Aortic Valve Replacement in Dialysis Patients: A Propensity-Matched Comparison. *Annals of Thoracic Surgery.* 2015. 100:1230-6; discussion 1236-7 |
|  | **D. Mesa,​ F. Castillo,​ M. Ruiz Ortiz,​ M. Puentes,​ J. Suarez de Lezo**. Impact of transcatheter aortic valve implantation or surgical aortic valve replacement on right ventricular function. *Heart.* 2013. 99:286 |
|  | **D. Wendt,​ F. Al-Rashid,​ P. Kahlert,​ K. El-Chilali,​ E. Demircioglu,​ M. Neuhauser,​ O. Liakopoulos,​ D. Sebastian Dohle,​ R. Erbel,​ H. Jakob,​ M. Thielmann**. Conventional aortic valve replacement or transcatheter aortic valve implantation in patients with previous cardiac surgery. *Journal of Cardiology.* 2015. 66:292-297 |
|  | **D. Wendt,​ M. Thielmann,​ P. Kahlert,​ S. Kastner,​ V. Price,​ F. Al-Rashid,​ P. Patsalis,​ R. Erbel,​ H. Jakob**. Comparison between different risk scoring algorithms on isolated conventional or transcatheter aortic valve replacement. *Annals of Thoracic Surgery.* 2014. 97:796-802 |
|  | **E. Im,​ M. K. Hong,​ Y. G. Ko,​ D. H. Shin,​ J. S. Kim,​ B. K. Kim,​ D. Choi,​ C. Y. Shim,​ H. J. Chang,​ J. K. Shim,​ Y. L. Kwak,​ S. Lee,​ B. C. Chang,​ Y. Jang**. Comparison of early clinical outcomes following transcatheter aortic valve implantation versus surgical aortic valve replacement versus optimal medical therapy in patients older than 80 years with symptomatic severe aortic stenosis. *Yonsei Medical Journal.* 2013. 54:596-602 |
|  | **E. L. Hannan,​ Z. Samadashvili,​ N. J. Stamato,​ S. J. Lahey,​ A. Wechsler,​ D. Jordan,​ T. M. Sundt,​ J. P. Gold,​ C. E. Ruiz,​ M. H. Ashraf,​ C. R. Smith**. Utilization and 1-Year Mortality for Transcatheter Aortic Valve Replacement and Surgical Aortic Valve Replacement in New York Patients With Aortic Stenosis: 2011 to 2012. *Jacc: Cardiovascular Interventions.* 2016. 9:578-85 |
|  | **F. Bauer,​ V. Coutant,​ M. Bernard,​ D. Stepowski,​ C. Tron,​ A. Cribier,​ J. P. Bessou,​ H. Eltchaninoff**. Patients with severe aortic stenosis and reduced ejection fraction: earlier recovery of left ventricular systolic function after transcatheter aortic valve implantation compared with surgical valve replacement. *Echocardiography.* 2013. 30:865-70 |
|  | **Francesco Onorati,​ Augusto D'Onofrio,​ Fausto Biancari,​ Stefano Salizzoni,​ Marisa De Feo,​ Marco Agrifoglio,​ Giovanni Mariscalco,​ Vincenzo Lucchetti,​ Antonio Messina,​ Francesco Musumeci,​ Giuseppe Santarpino,​ Giampiero Esposito,​ Francesco Santini,​ Paolo Magagna,​ Cesare Beghi,​ Marco Aiello,​ Ester Dalla Ratta,​ Carlo Savini,​ Giovanni Troise,​ Mauro Cassese,​ Theodor Fischlein,​ Mattia Glauber,​ Giancarlo Passerone,​ Giuseppe Punta,​ Tatu Juvonen,​ Ottavio Alfieri,​ Davide Gabbieri,​ Domenico Mangino,​ Andrea Agostinelli,​ Ugolino Livi,​ Omar Di Gregorio,​ Alessandro Minati,​ Mauro Rinaldi,​ Gino Gerosa,​ Giuseppe Faggian,​Record,​ I. T. A. investigators**. Results of surgical aortic valve replacement and transapical transcatheter aortic valve replacement in patients with previous coronary artery bypass grafting. *Interactive Cardiovascular & Thoracic Surgery.* 2016. 22:806-12 |
|  | **Francesco Onorati,​ Augusto D'Onofrio,​ Fausto Biancari,​ Stefano Salizzoni,​ Marisa De Feo,​ Marco Agrifoglio,​ Giovanni Mariscalco,​ Vincenzo Lucchetti,​ Antonio Messina,​ Francesco Musumeci,​ Giuseppe Santarpino,​ Giampiero Esposito,​ Francesco Santini,​ Paolo Magagna,​ Cesare Beghi,​ Marco Aiello,​ Ester Della Ratta,​ Carlo Savini,​ Giovanni Troise,​ Mauro Cassese,​ Theodor Fischlein,​ Mattia Glauber,​ Giancarlo Passerone,​ Giuseppe Punta,​ Tatu Juvonen,​ Ottavio Alfieri,​ Davide Gabbieri,​ Domenico Mangino,​ Andrea Agostinelli,​ Ugolino Livi,​ Omar Di Gregorio,​ Alessandro Minati,​ Mauro Rinaldi,​ Gino Gerosa,​ Giuseppe Faggian The Record Ita Investigators**. Aortic Valve Replacement in Redo-Scenarios: A Comparison Between Traditional Aortic Valve Replacement (TAVR) and Transapical-TAVR from Two Real-World Multicenter Registries. *Journal of Heart Valve Disease.* 2015. 24:669-678 |
|  | **G. Erdoes,​ C. Lippuner,​ I. Kocsis,​ M. Schiff,​ M. Stucki,​ T. Carrel,​ S. Windecker,​ B. Eberle,​ F. Stueber,​ M. Book**. Technical approach determines inflammatory response after surgical and transcatheter aortic valve replacement. *PLoS ONE.* 2015. 10 (11) (no pagination):#pages# |
|  | **G. Schymik,​ M. Heimeshoff,​ P. Bramlage,​ T. Herbinger,​ A. Wurth,​ L. Pilz,​ J. S. Schymik,​ R. Wondraschek,​ T. Suselbeck,​ J. Gerhardus,​ A. Luik,​ B. D. Gonska,​ P. Tzamalis,​ H. Posival,​ C. Schmitt,​ H. Schrofel**. A comparison of transcatheter aortic valve implantation and surgical aortic valve replacement in 1,​141 patients with severe symptomatic aortic stenosis and less than high risk. *Catheterization & Cardiovascular Interventions.* 2015. 86:738-44 |
|  | **H. Fox,​ S. Buttner,​ K. Hemmann,​ A. Asbe-Vollkopf,​ M. Doss,​ A. Beiras-Fernandez,​ A. Moritz,​ A. M. Zeiher,​ E. Scheuermann,​ H. Geiger,​ S. Fichtlscherer,​ I. A. Hauser,​ R. Lehmann**. Transcatheter aortic valve implantation improves outcome compared to open-heart surgery in kidney transplant recipients requiring aortic valve replacement. *Journal of Cardiology.* 2013. 61:423-7 |
|  | **Hardy Baumbach,​ Samir Ahad,​ Christian Rustenbach,​ Stephan Hill,​ Tim Schaufele,​ Kristina Wachter,​ Ulrich Friedrich Wilhelm Franke**. Conventional versus Transapical Aortic Valve Replacement: Is It Time for Shift in Indications?. *Thoracic & Cardiovascular Surgeon.* 2017. 65:212-217 |
|  | **I. Zweng,​ W. Y. Shi,​ S. Palmer,​ A. MacIsaac,​ R. Whitbourn,​ P. Davis,​ A. E. Newcomb**. Transcatheter versus Surgical Aortic Valve Replacement in High-risk Patients: A propensity-score matched analysis. *Heart Lung and Circulation.* 2016. 25:661-667 |
|  | **J. E. Davies,​ W. W. McAlexander,​ M. F. Sasse,​ M. A. Leesar,​ S. J. Melby,​ S. P. Singh,​ L. B. Jernigan,​ O. J. Booker,​ O. O. Alli**. Impact of Transcatheter Aortic Valve Replacement on Surgical Volumes and Outcomes in a Tertiary Academic Cardiac Surgical Practice. *Journal of the American College of Surgeons.* 2016. 222:645-55 |
|  | **J. K. Pettet,​ M. N. McGhee,​ S. T. McIlrath,​ G. L. Collins**. Comparison of pulmonary complications in patients undergoing transcatheter aortic valve implantation versus open aortic valve replacement. *Journal of Cardiothoracic & Vascular Anesthesia.* 2014. 28:497-501 |
|  | **J. Sulzenko,​ P. Tousek,​ V. Kocka,​ F. Bednar,​ H. Linkova,​ R. Petr,​ M. Labos,​ P. Widimsky**. Degenerative changes and immune response after transcatheter aortic valve implantation. Comparison with surgical aortic valve replacement. *Journal of Cardiology.* 2017. 69:483-488 |
|  | **J. T. Strauch,​ M. Scherner,​ P. L. Haldenwang,​ N. Madershahian,​ R. Pfister,​ E. W. Kuhn,​ O. J. Liakopoulos,​ J. Wippermann,​ T. Wahlers**. Transapical minimally invasive aortic valve implantation and conventional aortic valve replacement in octogenarians. *Thoracic & Cardiovascular Surgeon.* 2012. 60:335-42 |
|  | **Karin Olsson,​ Johan Nilsson,​ Asa Hornsten,​ Ulf Naslund**. Patients' self-reported function,​ symptoms and health-related quality of life before and 6 months after transcatheter aortic valve implantation and surgical aortic valve replacement. *European Journal of Cardiovascular Nursing.* 2017. 16:213-221 |
|  | **L. Conradi,​ M. Seiffert,​ H. Treede,​ M. Silaschi,​ S. Baldus,​ J. Schirmer,​ J. F. Kersten,​ T. Meinertz,​ H. Reichenspurner**. Transcatheter aortic valve implantation versus surgical aortic valve replacement: a propensity score analysis in patients at high surgical risk. *Journal of Thoracic & Cardiovascular Surgery.* 2012. 143:64-71 |
|  | **Laura E. Dobson,​ Tarique A. Musa,​ Akhlaque Uddin,​ Timothy A. Fairbairn,​ Peter P. Swoboda,​ David P. Ripley,​ Pankaj Garg,​ Betsy Evans,​ Christopher J. Malkin,​ Daniel J. Blackman,​ Sven Plein,​ John P. Greenwood**. Post-procedural myocardial infaRCTion following surgical aortic valve replacement and transcatheter aortic valve implantation. *Eurointervention.* 2017. 13:e153-e160 |
|  | **Luise Gaede,​ Johannes Blumenstein,​ Won-Keun Kim,​ Christoph Liebetrau,​ Oliver Dorr,​ Holger Nef,​ Christian Hamm,​ Albrecht Elsasser,​ Helge Mollmann**. Trends in aortic valve replacement in Germany in 2015: transcatheter versus isolated surgical aortic valve repair. *Clinical Research in Cardiology.* 2017. 106:411-419 |
|  | **M. A. Clavel,​ J. G. Webb,​ J. Rodes-Cabau,​ J. B. Masson,​ E. Dumont,​ R. De Larochelliere,​ D. Doyle,​ S. Bergeron,​ H. Baumgartner,​ I. G. Burwash,​ J. G. Dumesnil,​ G. Mundigler,​ R. Moss,​ A. Kempny,​ R. Bagur,​ J. Bergler-Klein,​ R. Gurvitch,​ P. Mathieu,​ P. Pibarot**. Comparison between transcatheter and surgical prosthetic valve implantation in patients with severe aortic stenosis and reduced left ventricular ejection fraction. *Circulation.* 2010. 122:1928-36 |
|  | **M. A. Sherif,​ M. Abdel-Wahab,​ O. Awad,​ V. Geist,​ G. El-Shahed,​ R. Semmler,​ M. Tawfik,​ A. A. Khattab,​ D. Richardt,​ G. Richardt,​ R. Tolg**. Early hemodynamic and neurohormonal response after transcatheter aortic valve implantation. *American Heart Journal.* 2010. 160:862-9 |
|  | **M. C. Henn,​ A. Zajarias,​ B. R. Lindman,​ J. W. Greenberg,​ S. J. Melby,​ N. Quader,​ A. M. Vatterott,​ C. Lawler,​ M. S. Damiano,​ E. Novak,​ J. M. Lasala,​ M. R. Moon,​ J. S. Lawton,​ R. J. Damiano,​ H. S. Maniar**. Preoperative pulmonary function tests predict mortality after surgical or transcatheter aortic valve replacement. *Journal of Thoracic & Cardiovascular Surgery.* 2016. 151:578-85,​ 586.e1-2 |
|  | **M. C. Mack,​ M. Szerlip,​ M. A. Herbert,​ S. Akram,​ C. Worley,​ R. J. Kim,​ B. A. Prince,​ K. B. Harrington,​ M. J. Mack,​ E. M. Holper**. Outcomes of Treatment of Nonagenarians With Severe Aortic Stenosis. *Annals of Thoracic Surgery.* 2015. 100:74-80 |
|  | **M. De Carlo,​ C. Giannini,​ F. Ettori,​ C. Fiorina,​ F. Guarracino,​ S. Curello,​ G. Scioti,​ G. Minzioni,​ G. Chizzola,​ D. Matteo,​ A. S. Petronio**. Impact of treatment choice on the outcome of patients proposed for transcatheter aortic valve implantation. *Eurointervention.* 2010. 6:568-74 |
|  | **M. Falcone,​ A. Russo,​ M. Mancone,​ G. Carriero,​ G. Mazzesi,​ F. Miraldi,​ M. Pennacchi,​ F. Pugliese,​ L. Tritapepe,​ V. Vullo,​ F. Fedele,​ G. Sardella,​ M. Venditti**. Early,​ intermediate and late infectious complications after transcatheter or surgical aortic-valve replacement: a prospective cohort study. *Clinical Microbiology & Infection.* 2014. 20:758-63 |
|  | **M. Johansson,​ S. Nozohoor,​ H. Bjursten,​ S. Ragnarsson,​ M. Gotberg,​ P. O. Kimblad,​ I. Zindovic,​ J. Sjogren**. Late survival and heart failure after transcatheter aortic valve implantation. *Asian Cardiovascular & Thoracic Annals.* 2016. 24:318-25 |
|  | **M. Scherner,​ N. Madershahian,​ K. Kuhr,​ S. Rosenkranz,​ E. Stoger,​ P. Rahmanian,​ Y. Choi,​ I. Slottosch,​ J. Wippermann,​ J. Strauch,​ T. Wahlers**. Aortic valve replacement after previous heart surgery in high-risk patients: transapical aortic valve implantation versus conventional aortic valve replacement-a risk-adjusted and propensity score-based analysis. *Journal of Thoracic & Cardiovascular Surgery.* 2014. 148:90-7 |
|  | **M. Wilbring,​ S. M. Tugtekin,​ K. Alexiou,​ G. Simonis,​ K. Matschke,​ U. Kappert**. Transapical transcatheter aortic valve implantation vs conventional aortic valve replacement in high-risk patients with previous cardiac surgery: a propensity-score analysis. *European Journal of Cardio-Thoracic Surgery.* 2013. 44:42-7 |
|  | **Michael Salna,​ Omar K. Khalique,​ Codruta Chiuzan,​ Paul Kurlansky,​ Michael A. Borger,​ Rebecca T. Hahn,​ Martin B. Leon,​ Craig R. Smith,​ Susheel K. Kodali,​ Isaac George**. Impact of small prosthesis size on transcatheter or surgical aortic valve replacement outcomes. *Catheterization & Cardiovascular Interventions.* 2017. 04:04 |
|  | **N. Papadopoulos,​ N. Schiller,​ S. Fichtlscherer,​ R. Lehmann,​ C. F. Weber,​ A. Moritz,​ M. Doss,​ A. Zierer**. Propensity matched analysis of longterm outcomes following transcatheter based aortic valve implantation versus classic aortic valve replacement in patients with previous cardiac surgery. *Journal Of Cardiothoracic Surgery.* 2014. 9:99 |
|  | **N. Piazza,​ B. Kalesan,​ N. van Mieghem,​ S. Head,​ P. Wenaweser,​ T. P. Carrel,​ S. Bleiziffer,​ P. P. de Jaegere,​ B. Gahl,​ R. H. Anderson,​ A. P. Kappetein,​ R. Lange,​ P. W. Serruys,​ S. Windecker,​ P. Juni**. A 3-center comparison of 1-year mortality outcomes between transcatheter aortic valve implantation and surgical aortic valve replacement on the basis of propensity score matching among intermediate-risk surgical patients. *Jacc: Cardiovascular Interventions.* 2013. 6:443-51 |
|  | **P. D'Errigo,​ M. Barbanti,​ F. Santini,​ C. Grossi,​ M. Ranucci,​ F. Onorati,​ R. D. Covello,​ S. Rosato,​ C. Tamburino,​ G. Santoro,​ D. Fusco,​ F. Seccareccia,​ Observant Gruppo di Lavoro dello Studio**. [Results of the OBSERVANT study: clinical characteristics and short-term outcome of the enrolled population treated with transcatheter versus surgical aortic valve implantation]. *Giornale Italiano di Cardiologia.* 2014. 15:177-84 |
|  | **P. Kala,​ M. Tretina,​ M. Poloczek,​ J. Ondrasek,​ P. Malik,​ P. Pokorny,​ J. Parenica,​ J. Spinar,​ J. Jarkovsky,​ S. Littnerova,​ P. Nemec**. Quality of life after transcatheter aortic valve implantation and surgical replacement in high-risk elderly patients. *Biomedical Papers of the Medical Faculty of Palacky University in Olomouc,​ Czech Republic.* 2013. 157:75-80 |
|  | **P. Wenaweser,​ T. Pilgrim,​ A. Kadner,​ C. Huber,​ S. Stortecky,​ L. Buellesfeld,​ A. A. Khattab,​ F. Meuli,​ N. Roth,​ B. Eberle,​ G. Erdos,​ H. Brinks,​ B. Kalesan,​ B. Meier,​ P. Jni,​ T. Carrel,​ S. Windecker**. Clinical outcomes of patients with severe aortic stenosis at increased surgical risk according to treatment modality. *Journal of the American College of Cardiology.* 2011. 58:2151-2162 |
|  | **Phillip J. Tully,​ Prakash Roshan,​ Greg D. Rice,​ Ajay Sinhal,​ Jayme S. Bennetts,​ Robert A. Baker**. Change in quality of life after transcatheter aortic valve implantation and aortic valve replacement surgery in Australian patients aged >= 75 years: the effects of EuroSCORE and patient operability. *Journal of Geriatric Cardiology.* 2015. 12:30-6 |
|  | **Prakash Balan,​ Yelin Zhao,​ Sarah Johnson,​ Salman Arain,​ Abhijeet Dhoble,​ Anthony Estrera,​ Richard Smalling,​ Tom C. Nguyen**. The Society of Thoracic Surgery Risk Score as a Predictor of 30-Day Mortality in Transcatheter vs Surgical Aortic Valve Replacement: A Single-Center Experience and its Implications for the Development of a TAVR Risk-Prediction Model. *Journal of Invasive Cardiology.* 2017. 29:109-114 |
|  | **R. J. Nuis,​ A. E. Dager,​ R. M. van der Boon,​ M. C. Jaimes,​ B. Caicedo,​ J. Fonseca,​ N. M. Van Mieghem,​ L. M. Benitez,​ J. P. Umana,​ W. W. O'Neill,​ E. de Marchena,​ P. P. de Jaegere**. Patients with aortic stenosis referred for TAVI: treatment decision,​ in-hospital outcome and determinants of survival. *Netherlands Heart Journal.* 2012. 20:16-23 |
|  | **R. L. Osnabrugge,​ S. J. Head,​ T. S. Genders,​ N. M. Van Mieghem,​ P. P. De Jaegere,​ R. M. van der Boon,​ J. M. Kerkvliet,​ B. Kalesan,​ A. J. Bogers,​ A. P. Kappetein,​ M. G. Hunink**. Costs of transcatheter versus surgical aortic valve replacement in intermediate-risk patients. *Annals of Thoracic Surgery.* 2012. 94:1954-60 |
|  | **R. Stohr,​ G. Dohmen,​ R. Herpertz,​ K. Brehmer,​ O. Aktug,​ R. Koos,​ E. Altiok,​ E. Stegemann,​ R. Autschbach,​ N. Marx,​ R. Hoffmann**. Thirty-day outcome after transcatheter aortic valve implantation compared with surgical valve replacement in patients with high-risk aortic stenosis: a matched comparison. *Coronary Artery Disease.* 2011. 22:595-600 |
|  | **Ryosuke Higuchi,​ Tetsuya Tobaru,​ Kenichi Hagiya,​ Mike Saji,​ Keitaro Mahara,​ Itaru Takamisawa,​ Jun Shimizu,​ Shuichiro Takanashi,​ Morimasa Takayama**. Transcatheter aortic valve implantation in patients on corticosteroid therapy. *Heart & Vessels.* 2017. 23:23 |
|  | **S. Rau,​ M. Wessely,​ P. Lange,​ C. Kupatt,​ G. Steinbeck,​ M. Fischereder,​ U. Schnermarck**. Transcatheter aortic valve implantation in dialysis patients. *Nephron - Clinical Practice.* 2012. 120:c86-c90 |
|  | **S. Silberman,​ F. Abu Akr,​ D. Bitran,​ Y. Almagor,​ J. Balkin,​ R. Tauber,​ O. Merin**. Comparison between transcatheter and surgical aortic valve replacement: a single-center experience. *Journal of Heart Valve Disease.* 2013. 22:448-54 |
|  | **S. W. Grant,​ G. L. Hickey,​ P. Ludman,​ N. Moat,​ D. Cunningham,​ M. de Belder,​ D. J. Blackman,​ D. Hildick-Smith,​ R. Uppal,​ S. Kendall,​ B. Bridgewater**. Activity and outcomes for aortic valve implantations performed in England and Wales since the introduction of transcatheter aortic valve implantation. *European Journal of Cardio-Thoracic Surgery.* 2016. 49:1164-73 |
|  | **S. W. Grant,​ M. P. Devbhandari,​ A. D. Grayson,​ I. Dimarakis,​ I. Kadir,​ D. M. Saravanan,​ R. D. Levy,​ S. G. Ray,​ B. Bridgewater**. What is the impact of providing a transcatheter aortic valve implantation service on conventional aortic valve surgical activity: patient risk factors and outcomes in the first 2 years. *Heart.* 2010. 96:1633-7 |
|  | **Seyed Hossein Aalaei-Andabili,​ R. David Anderson,​ John W. Petersen,​ Thomas M. Beaver,​ Anthony A. Bavry,​ Charles T. Klodell**. Comparison of periprocedural and mid-term stroke rates and outcomes between surgical aortic valve replacement and transcatheter aortic valve replacement patients. *Journal of Cardiovascular Surgery.* 2017. 58:591-597 |
|  | **T. A. Fairbairn,​ C. D. Steadman,​ A. N. Mather,​ M. Motwani,​ D. J. Blackman,​ S. Plein,​ G. P. McCann,​ J. P. Greenwood**. Assessment of valve haemodynamics,​ reverse ventricular remodelling and myocardial fibrosis following transcatheter aortic valve implantation compared to surgical aortic valve replacement: a cardiovascular magnetic resonance study. *Heart.* 2013. 99:1185-91 |
|  | **T. A. Mabin,​ P. Condolfi**. An analysis of real-world cost-effectiveness of TAVI in South Africa. *Cardiovascular Journal of Africa.* 2014. 25:21-6 |
|  | **T. Ando,​ E. Akintoye,​ T. Telila,​ A. Briasoulis,​ H. Takagi,​ D. P. Slovut,​ T. Schreiber,​ C. L. Grines,​ L. Afonso**. Comparison of Hospital Outcome of Transcatheter Versus Surgical Aortic Valve Replacement in Patients With Diabetes Mellitus (from the Nationwide Inpatient Sample). *American Journal of Cardiology.* 2017. 119:1250-1254 |
|  | **T. C. Nguyen,​ V. C. Babaliaros,​ S. A. Razavi,​ P. D. Kilgo,​ R. A. Guyton,​ C. M. Devireddy,​ C. C. Shults,​ K. Mavromatis,​ M. Kanitkar,​ P. Block,​ S. Lerakis,​ V. H. Thourani**. Impact of varying degrees of renal dysfunction on transcatheter and surgical aortic valve replacement. *Journal of Thoracic & Cardiovascular Surgery.* 2013. 146:1399-406; discussion 13406-7 |
|  | **T. K. Steigen,​ B. Schive,​ T. Naesheim,​ R. Busund**. Transcatheter aortic-valve implantation for aortic stenosis. *Tidsskrift for Den Norske Laegeforening.* 2011. 131:343-8 |
|  | **T. M. Dewey,​ D. L. Brown,​ T. S. Das,​ W. H. Ryan,​ J. E. Fowler,​ S. D. Hoffman,​ S. L. Prince,​ M. A. Herbert,​ D. Culica,​ M. J. Mack**. High-risk patients referred for transcatheter aortic valve implantation: management and outcomes. *Annals of Thoracic Surgery.* 2008. 86:1450-6; discussion 1456-7 |
|  | **T. Murashita,​ K. L. Greason,​ R. M. Suri,​ V. T. Nkomo,​ D. R. Holmes,​ C. S. Rihal,​ V. Mathew**. Aortic valve replacement for severe aortic valve stenosis in the nonagenarian patient. *Annals of Thoracic Surgery.* 2014. 98:1593-7 |
|  | **T. Tokarek,​ R. Sobczynski,​ A. Dziewierz,​ Z. Siudak,​ W. Zasada,​ D. Sorysz,​ R. Pfitzner,​ J. Sadowski,​ G. Debski,​ E. Dziewiecka,​ K. Gruszka,​ D. Dudek**. Clinical outcomes in patients after surgical and transcatheter aortic valve replacement. *Polskie Archiwum Medycyny Wewnetrznej.* 2015. 125:755-764 |
|  | **T. Walther,​ G. Schuler,​ M. A. Borger,​ J. Kempfert,​ J. Seeburger,​ Y. Ruckert,​ J. Ender,​ A. Linke,​ M. Scholz,​ V. Falk,​ F. W. Mohr**. Transapical aortic valve implantation in 100 consecutive patients: comparison to propensity-matched conventional aortic valve replacement. *European Heart Journal.* 2010. 31:1398-403 |
|  | **Tarique Al Musa,​ Akhlaque Uddin,​ Timothy A. Fairbairn,​ Laura E. Dobson,​ Christopher D. Steadman,​ Ananth Kidambi,​ David P. Ripley,​ Peter P. Swoboda,​ Adam K. McDiarmid,​ Bara Erhayiem,​ Pankaj Garg,​ Daniel J. Blackman,​ Sven Plein,​ Gerald P. McCann,​ John P. Greenwood**. Right ventricular function following surgical aortic valve replacement and transcatheter aortic valve implantation: A cardiovascular MR study. *International Journal of Cardiology.* 2016. 223:639-644 |
|  | **Tom C. Nguyen,​ Matthew D. Terwelp,​ Vinod H. Thourani,​ Yelin Zhao,​ Nidal Ganim,​ Carson Hoffmann,​ Monica Justo,​ Anthony L. Estrera,​ Richard W. Smalling,​ Prakash Balan,​ Joseph Lamelas**. Clinical trends in surgical,​ minimally invasive and transcatheter aortic valve replacement+. *European Journal of Cardio-Thoracic Surgery.* 2017. 51:1086-1092 |
|  | **V. Bhise,​ P. Kanade,​ G. P. S. Shantha,​ P. Balan,​ T. C. Nguyen,​ P. Loyalka,​ B. Kar,​ A. Estrera,​ R. W. Smalling,​ A. Dhoble**. Transcatheter and Surgical Aortic Valve Replacement in Patients With End-Stage Renal Disease. *Journal of the American College of Cardiology.* 2017. 69:1875-1876 |
|  | **W. Awad,​ A. Mathur,​ L. Baldock,​ S. Oliver,​ S. Kennon**. Comparing post-operative resource consumption following transcatheter aortic valve implantation (TAVI) and conventional aortic valve replacement in the UK. *Journal of Medical Economics.* 2014. 17:357-64 |
|  | **Woo Sik Yu,​ Byung-Chul Chang,​ Hyun Chel Joo,​ Young-Guk Ko,​ Sak Lee**. Comparison of Early Clinical Results of Transcatheter versus Surgical Aortic Valve Replacement in Symptomatic High Risk Severe Aortic Stenosis Patients. *The Korean Journal of Thoracic & Cardiovascular Surgery.* 2013. 46:346-52 |
|  | **Y. Q. Ye,​ Y. T. Wang,​ Z. Li,​ M. Y. Wang,​ H. Y. Xu,​ W. J. Zhang,​ Q. R. Liu,​ G. N. Niu,​ Y. J. Wu**. [Outcome comparison of different therapy procedures in surgical high-risk elderly patients with severe aortic stenosis]. *Chung-Hua Hsin Hsueh Kuan Ping Tsa Chih [Chinese Journal of Cardiology].* 2017. 45:13-18 |

**Table S4**. Cochrane Risk Of Bias (ROB) assessment of RCTs

| Study | Random sequence generated | Mechanism of allocation concealed | Blinded participants/ personnel to allocation | Blinded outcome assessment to allocation | Incomplete outcome addressed | Free of selective reporting | Free of other biases | Total | Quality of trial |
| --- | --- | --- | --- | --- | --- | --- | --- | --- | --- |
| PARTNER1A (2011) | Yes | Yes | No | Yes | Yes | Yes | Yes | 6 | high |
| PARTNER2A (2016) | Yes | Yes | No | Yes | Yes | Yes | Yes | 6 | high |
| NOTION (2016) | Yes | Yes | No | Yes | Yes | Yes | Yes | 6 | high |
| USCV (2014) | Unclear | Yes | No | Unclear | Yes | Yes | Yes | 4 | low |
| SURTAVI (2017) | Unclear | Yes | No | Unclear | Yes | Yes | Yes | 4 | low |
| STACCATO* (2012) | Unclear | Unclear | Unclear | Unclear | Unclear | No | Unclear | 0 | low |

A trial was determined to be of high quality if all attributes except blinding of study subjects were deemed adequate. Dates indicate year of first publication from each trial.

* This trial was terminated prematurely due to safety concerns. The report did not yield enough information to evaluate all areas
